# Novel protein complexes containing autophagy and UPS components regulate proteasome-dependent PARK2 recruitment onto mitochondria and PARK2-PARK6 activity during mitophagy

**DOI:** 10.1038/s41419-022-05339-x

**Published:** 2022-11-10

**Authors:** Nur Mehpare Kocaturk, Nesibe Peker, Karin Eberhart, Yunus Akkoc, Gamze Deveci, Joern Dengjel, Devrim Gozuacik

**Affiliations:** 1 SUNUM Nanotechnology Research and Application Center, Istanbul, Turkey; 2 Koç University Research Center for Translational Medicine (KUTTAM), Istanbul, Turkey; 3 University of Fribourg, Fribourg, Switzerland; 4 Koç University School of Medicine, Istanbul, Turkey; 5 Present Address: School of Life Sciences, University of Dundee, Dundee, UK

**Keywords:** Mitophagy, Macroautophagy

## Abstract

Autophagy is an evolutionarily conserved eukaryotic cellular mechanism through which cytosolic fragments, misfolded/aggregated proteins and organelles are degraded and recycled. Priming of mitochondria through ubiquitylation is required for the clearance the organelle by autophagy (mitophagy). Familial Parkinson’s Disease-related proteins, including the E3-ligase PARK2 (PARKIN) and the serine/threonine kinase PARK6 (PINK1) control these ubiquitylation reactions and contribute to the regulation of mitophagy. Here we describe, novel protein complexes containing autophagy protein ATG5 and ubiquitin-proteasome system (UPS) components. We discovered that ATG5 interacts with PSMA7 and PARK2 upon mitochondrial stress. Results suggest that all three proteins translocate mitochondria and involve in protein complexes containing autophagy, UPS and mitophagy proteins. Interestingly, PARK2 and ATG5 recruitment onto mitochondria requires proteasome components PSMA7 and PSMB5. Strikingly, we discovered that subunit of 20 S proteasome, PSMA7, is required for the progression of PARK2-PARK6-mediated mitophagy and the proteasome activity following mitochondrial stress. Our results demonstrate direct, dynamic and functional interactions between autophagy and UPS components that contribute to the regulation of mitophagy.

## Introduction

Autophagy is a highly conserved degradation process during which targets are engulfed in double-memsbrane autophagosomes (autophagic vesicles), and degraded in autolysosomes that are formed following autophagosome-lysosome fusion [1, 2]. Autophagy determines the half-life of long-lived proteins and organelles, eliminates protein aggregates and destroys intracellular invaders. Moreover, as one of the primary stress response, autophagy allows survival of cells under detrimental conditions, such as nutrient and growth factor deprivation, oxidative stress and exposure to drugs and toxins. Autolysosomal degradation of cargoes into their basic components (e.g. proteins to amino acids) supplies building blocks for cellular functions, allowing synthesis of macromolecules and production of energy under stress conditions.

To date, more than 30 different core AuTophagy-related Genes (ATG proteins) were characterized [3]. These proteins form complexes and catalyse autophagic reactions. Especially two ubiquitylation-like reactions are key for the elongation of autophagic membranes: The first ubiquitylation-like reaction leads to the covalent conjugation of the ATG5 protein to a ubiquitin-like protein, ATG12. Next, ATG16L1 joins ATG12-ATG5, contributing to the formation of a higher molecular weight protein complex of around 669-800 kDa [4, 5]. ATG12-ATG5-ATG16L1 complex serves as an E3-like enzyme for the second ubiquitylation-like reaction. As third reaction, covalent attachment of ATG8/LC3 proteins to lipid molecules, generally to a phosphatidylethanolamine (PE), results in the elongation and closure of autophagic membranes [6, 7]. ATG12-ATG5-ATG16L1 and LC3 conjugation systems are indispensable for the canonical autophagy pathway, and their defects result in autophagy abnormalities in cells and mice [8].

Autophagy has long been considered as a non-selective degradation process. Previous studies demonstrated that selective autophagy exists [9, 10], and involves targeted sequestration of molecules and organelles by autophagy receptors, such as sequestosome 1 (P62/SQSTM1), optineurin (OPTN), calcium binding and coiled-coil domain 2 (CALCOCO2), NBR1 autophagy cargo receptor (NBR1), Tax1 binding protein 1 (TAX1BP1) etc. Selective autophagy of mitochondria (mitophagy) is the main cellular process that eliminates and recycles aberrant, depolarized, dysfunctional and/or damaged mitochondria. Hence, mitophagy serves as a major organelle abundance and quality control mechanism in all eukaryotic cells [11, 12].

Priming of mitochondria by the ubiquitin-proteasome system (UPS) is required for the progression of mitophagy. The UPS involves poly-ubiquitylation of target proteins and their degradation through the proteasomes [13]. Poly-ubiquitylation reactions are mediated by E1 (Ubiquitin activating), E2 (Ubiquitin conjugating) and E3 (Ubiquitin ligase) enzymes. The 26 S proteasome consists of the 19 S cap that mainly functions in the recruitment of the proteins to the proteasomes whereas 20 S proteasome is responsible for proteolytic degradation [14]. The 20 S subunit consists of several α and ß subunits. The α subunits lack catalytic activity and they mainly regulate entrance and exit of target proteins along the catalytic tunnel that consists of ß subunits. Chymotrypsin-like, caspase-like and trypsin-like proteolytic activities of the ß subunits cleave proteins into small polypeptides and amino acids, allowing their recycling for reuse. Among the α subunits, α7 (also known as PSMA7, RC6-1 or XACP7), is one of the best characterized. In addition to its core that participates in the structure of the 20 S proteasome, PSMA7 contains a protruding C-terminus that is available for protein-protein interactions. PSMA7 has been implicated in cancer [15, 16] and amyotrophic lateral sclerosis (ALS) [17], yet its mechanism of action remains to be elucidated in those models.

At least two familial Parkinson’s Disease-associated genes, namely the E3-ligase *PARK2* (*PARKIN*) and *PARK6* (*PINK1*) were reported in the control of mitophagy. Following the synthesis in cytosol, the 63 kDa serine/threonine kinase, PARK6, is targeted to the mitochondrial inner membrane. Under normal conditions, processing of the protein by mitochondrial proteases mitochondrial processing peptidase (MPP) and presenilin associated rhomboid like (PARL) leads to its cleavage to a shorter 55 kDa form. The short form does not accumulate on mitochondria and retrotranslocates to cytosol for proteasomal degradation [18]. Mitochondrial stress and loss of mitochondrial membrane potential, result in the retention of the 63 kDa, uncleaved form of PARK6 on the outer mitochondrial membrane (OMM). PARK6 and other mitochondrial proteins, including VDAC1/2 were involved in the recruitment and accumulation of PARK2 on the OMM [19]. Moreover, it was reported that the long form of PARK6 phosphorylates ubiquitin proteins as well as PARK2, further promoting PARK2 translocation onto mitochondria and stimulating its E3 ubiquitin ligase activity [20–22]. Subsequent ubiquitylation of target proteins, such as translocase of outer mitochondria membrane 40 (TOMM40), mitofusin 1 (MFN1), mitofusin 2 (MFN2) and ras homology family member T1 (MIRO1) by PARK2 leads to degradation of these proteins [23–26]. Additionally, integral membrane proteins on mitochondria, such as FUN14 domain containing 1 (FUNDC1) and ubiquitin specific peptidase 14 (USP14) regulated Prohibitin2 (PHB2), serve as adaptor proteins for recruitment of autophagosomes via direct interaction with LC3 proteins [27–29].

Autophagy and UPS are two critical quality control systems and tightly regulated in cells. Given this, impaired autophagy and/or UPS have been implicated in various diseases, including cancer, metabolic diseases, aging, neurodegenerative diseases, and immune system disorders. Besides, these two mechanisms crosstalk through various cellular pathways and their regulation is controlled by intersecting pathways. Alteration in one mechanism results in compensation or inhibition of the other mechanism depending on the context [30]. For example, inhibition of autophagy was shown to compromise UPS activity, which in turn results in accumulation of short-lived and aggregation-prone proteins [31].

Recent studies shed light on various aspects of ubiquitylation and LC3 recruitment mechanisms during mitophagy. Previous studies have documented degradation of mitochondrial proteins in a proteasome-dependent manner, underlining the crosstalk between UPS and autophagic degradation of mitochondria [26, 32]. Proteasome facilitates the degradation of mitochondrial proteins residing in both OMM and inner mitochondrial membrane (IMM). Also, mitochondrial precursor proteins that fail to translocate to mitochondria are degraded through the activity of proteasome [33]. Besides, UPS ensures mitochondrial quality control and mitophagy by selectively removing fusion and fission components, including MFNs, dynamin 1 like (DRP1) and fission mitochondrial 1 (FIS1). Moreover, starvation-induced autophagy leads to ubiquitylation of UPS proteins, increasing the interaction of UPS with autophagy proteins LC3 and P62 [34]. Given these, UPS, autophagy and mitophagy are regulated through overlapping cellular pathways, yet the exact mechanisms and which players are involved in this crosstalk remains unstudied.

Next, we showed that, mitophagy-inducing stress stimuli promotes formation of large and dynamic protein complexes containing the UPS and autophagy components. We also showed that the key autophagy protein ATG5 physically interacts with PARK2 and the 20 S proteasome subunits PSMA7 and PSMB5, and they altogether translocate onto stressed mitochondria. This event and subsequent mitophagy rely on 20 S proteasome components as well as the proteasomal activity. Our results suggested that novel protein complexes are formed on mitochondria containing autophagy protein ATG5, mitophagy proteins PARK2 and PARK6, and UPS proteins PSMA7 and PSMB5. We discovered that PSMA7 and PSMB5 are required for PARK2-PARK6-mediated mitophagy. Moreover, our results suggested that knockdown of PSMA7 significantly impairs PARK2-PARK6 interaction and attenuates mitochondrial stress-induced PARK2 E3 ubiquitin ligase activity, and ubiquitin-dependent degradation of PARK2 targets. Our results also showed that ubiquitin-dependent mitophagy receptor optineurin recruitment is hindered, mitochondria-associated LC3 levels are reduced and mitophagy is inhibited under these conditions. Furthermore, although ATG5 deficiency did not affect PARK2 recruitment onto mitochondria, a notable reduction in ubiquitylation and ubiquitin phosphorylation was observed upon mitochondrial stress. Collectively, these results suggest that the PARK2/PARK6 system, autophagy and the proteasome are tightly connected, and their functions are coordinated during progression of mitophagy.

## Results

### Autophagy Protein ATG5 Interacts with the UPS Component PSMA7 and mitophagy protein PARK2 in Response to Mitochondrial Stress

**Fig. 1:**
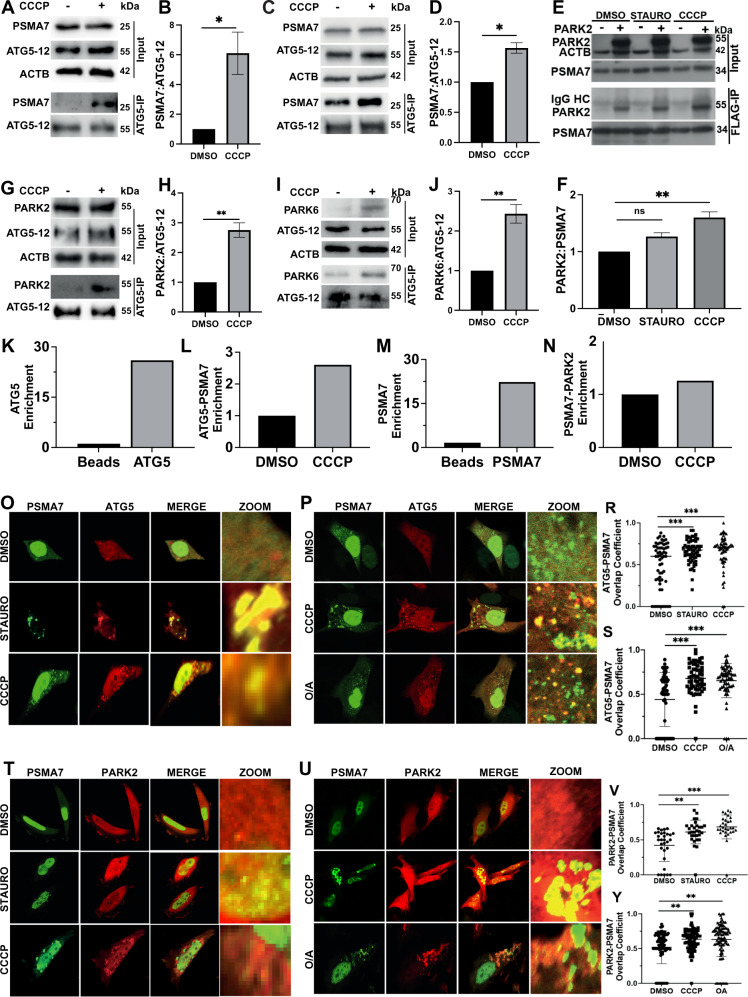
Mitochondrial stress promotes the interaction of autophagy and UPS components. **A** Representative western blot image showing endogenous PSMA7 and ATG5-12 protein levels in total cell lysate (Input) and ATG5-immunoprecipitated lysate (ATG5-IP) in HEK 293 T cells. Actin beta (ACTB) was used as loading control. **B** Graph representing the levels of immunoprecipitated PSMA7 protein with ATG5-12, normalized to ATG5-12 in HEK 293 T cells (mean ± SEM, *n* = 3). **C** Representative western blot image showing endogenous PSMA7 and ATG5-12 protein levels in total cell lysate (Input) and ATG5-immunoprecipitated lysate (ATG5-IP) in MEF cells. ACTB was used as loading control. **D** Graph representing the levels of immunoprecipitated PSMA7 protein with ATG5-12, normalized to ATG5-12 in MEF cells (mean ± SEM, *n* = 3). **E** Representative images of immunoprecipitation of FLAG and western blot analysis of PARK2 and PSMA7 in HEK 293 T cells co-transfected with MYC-PARK2 and/or FLAG-PSMA7. HEK 293 T cells were treated with STAURO (1 μM), CCCP (10 μM) or DMSO for 12 h after transfection and immunoprecipitation using Flag beads**. F** Quantification of PARK2 protein levels immunoprecipitated with PSMA7 from **E**, normalized to PSMA7 (mean ± SEM, *n* = 3). *G* and *I*, Representative western blot images showing the levels of PARK2 and PARK6 proteins respectively, immunoprecipitated with endogenous ATG5 in HA-PARK2 expressing HEK 293 T cells following DMSO or CCCP treatment (10 μM) for 12 h. **H** and **J**, Graphs showing the quantification of PARK2 (from **G**) and PARK6 (from **I**) protein levels, normalized to endogenous ATG5-12 (mean ± SEM, *n* = 3). **K** and **L**, SILAC-MS/MS-based interactome results as fold change graphs of ATG5 enrichment (**K**) and CCCP-induced PSMA7-ATG5 complex enrichment (*L*) as compared to control. *M* and *N*, Fold change graphs of PSMA7 enrichment (**M**) and CCCP-induced PARK2-PSMA7 complex enrichment (**N**). **O** and **P**, Confocal microscopy images of HEK 293 T cells co-transfected with GFP-PSMA7 (green) and pmCherry-ATG5 (red) constructs and treated with CCCP (10 μM) or STAURO for 12 h (**O**) and treated with CCCP (20 μM) or Oligomycin A/Antimycin A (O/A, 10 μM) for 2 h (**P**). **R** and **S**, overlap coefficiency graphs representing PSMA7 (green) and ATG5 (red) colocalization following 12 h (R) and 2 h (**S**) of treatments (*n* = 60). **T** and **U**, Confocal microscopy images of HeLa cells that were co-transfected with pEGFP-PSMA7 (green) and pmCherry-PARK2 (red) constructs, and treated with staurosporine (1 μM, 12 h) or CCCP (10 μM, 12 h) (**T**) and CCCP or O/A for 2 h (**U**). **V** and **Y**, Overlap coefficiency graphs representing PSMA7 (green) and PARK2 (red) colocalization after 12 h (**V**, *n* = 30) and 2 h (**Y**, *n* = 91) of treatments. MERGE, overlay of green and red signals. ZOOM, zoomed images of particular area. Significance in *R*, *S*, *V* and *Y* was determined using one-way ANOVA.

In order to discover new interaction partners of the autophagy protein ATG5, we performed unbiased classical yeast-two-hybrid screens [35]. One of the strong interactors of ATG5 was the 20 S proteasome component PSMA7 (C-term end of the protein, amino acids 174-248). We then confirmed this interaction through co-immunoprecipitation (Co-IP) experiments in HEK293T cells following overexpression of full-length proteins (Fig. S1A). Besides, we observed a notable increase in the interaction between ATG5-12 and PSMA7 proteins following mitochondrial stress (i.e., STAURO or CCCP treatment) (Fig. S1B). Interaction between endogenous ATG5-12 and PSMA7 proteins was barely detected under control condition. Strikingly, the interaction between endogenous ATG5-12 and PSMA7 significantly increased in HEK293T (Fig. 1A and B) and MEF (Fig. 1C and D) cells upon CCCP treatment. Similarly, our results revealed that ATG5 and other 20 S subunit protein, PSMB5, interacted upon mitochondrial depolarization (Fig. S1C). Damaged mitochondria are mainly cleared by mitophagy, and PARK2 was identified as a key E3 ubiquitin ligase regulator of this phenomenon. Interestingly, PARK2 was previously reported to interact with PSMA7/XAPC7 [36]. Therefore, we also checked this interaction under our experimental conditions. Indeed, PARK2 interacted with PSMA7 upon CCCP-induced mitochondrial stress, but STAURO failed to further stimulate this interaction (Fig. 1E and F). Strikingly, our results revealed that PARK2 interacts with ATG5-12 complex upon mitochondrial stress (Fig. 1G and H). Also, to explore whether endogenous PARK2 interacts with ATG5, we performed immunoprecipitation experiments using in HT-22 and SH-SY5Y cells in which endogenous PARK2 protein levels are detectable. Results further confirmed endogenous ATG5 and PARK2 interacts in HT-22 (Fig. S1D) and SH-SY5Y (Fig. S1E) cells. Also, results showed this interaction was enhanced following mitochondrial stress in HT-22 cells (Fig. S1D). In line with these, results showed the interaction of PARK2 partner protein, PARK6, with ATG5-12 complex, and increased interaction of these proteins upon mitochondrial stress (Fig. 1, I and J). SILAC-MS/MS experiments using ATG5 as a bait further confirmed the interaction with PSMA7 (Fig. 1K and L) and in a similar manner, when PSMA7 was used as a bait, interaction with PARK2 was enhanced under mitochondrial stress conditions (Fig. 1M and N) (Fig. 1K–N, fold change graphs representing the *n* = 1 experiments). Furthermore, confocal microscope analyses revealed increased colocalization of PSMA7-ATG5 under both long-term (12 h) stress caused by CCCP or STAURO (Fig. 1O and R) and short-term (2 h) stress caused by CCCP or Oligomycin/Antimycin A (O/A) (Fig. 1 P and S). Similarly, results also showed that CCCP- and STAURO-induced long term mitochondrial stress, and CCCP- and O/A-induced short term mitochondrial stress enhance the colocalization of PSMA7 with PARK2 (Fig. 1T–Y). Also, confocal images revealed that neither ATG5 (Fig. S1F and G) nor PARK2 (Fig. S1H and I) overexpression result in a dramatic change in the overall subcellular distribution of PSMA7. All together, these data suggest that PSMA7 interacts with ATG5 and PARK2 and this interaction is further enhanced upon mitochondrial stress.

### ATG5, PSMA7 and PARK2 migrated onto mitochondria under stress conditions

**Fig. 2:**
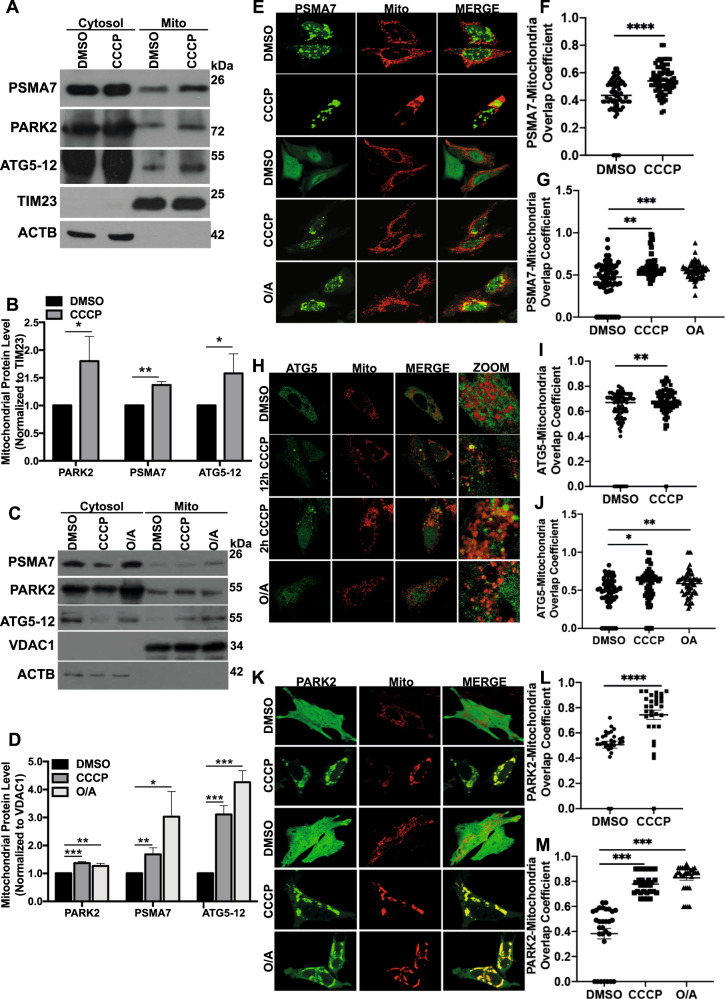
ATG5, PSMA7 and PARK2 translocated onto mitochondria upon mitochondrial stress. **A** Representative western blot images showing PSMA7, PARK2, ATG5-12, TIM23 and Actin beta (ACTB) protein levels in cytoplasmic (Cytosol) and mitochondrial (Mito) fractions of YFP-PARK2 transfected HEK 293 T cells treated with DMSO or CCCP (10 µM, 12 h). **B** Quantification graph corresponding to mitochondrial protein levels of PARK2, PSMA7 and ATG5-12 in **A**. Mitochondrial protein levels were normalized to TIM23 (mean ± S.D., *n* = 3). **C** Representative western blot images showing PSMA7, PARK2, ATG5-12, VDAC1, and ACTB protein levels in cytoplasmic (Cytosol) and mitochondrial (Mito) fractions of HA-PARK2 overexpressing HeLa cells treated with DMSO or CCCP (20 µM, 2 h) or O/A (10 µM, 2 h). **D** Quantification graph corresponding to protein levels of PARK2, PSMA7 and ATG5-12 in **C**. Mitochondrial protein levels were normalized to VDAC1 (mean ± S.D., *n* = 3). **E** Confocal microscopy analysis of HeLa cells co-transfected with pEGFP-PSMA7 (green) and mito-dsRed (red) constructs, and treated with DMSO or CCCP for 12 h (upper panel) and DMSO, CCCP or O/A for 2 h (lower panel). MERGE, overlay of green and red signals. **F** and **G** Overlap coefficiency graphs representing PSMA7 (green) and mitochondria (red) colocalization during 12 h (**F**, *n* = 68) and 2 h (**G**, *n* = 62) treatments. **H** Confocal microscopy analysis of HeLa cells co-transfected with YFP-ATG5 (green) and mito-dsRed (red) constructs and treated with DMSO or CCCP for 12 h (upper panel) and CCCP or O/A for 2 h (lower panel). **I** and **J** Overlap coefficiency graphs representing ATG5 (green) and mitochondria (red) colocalization during 12 h (**I**, *n* = 87) and 2 h (**J**, *n* = 60) treatments. **K** Confocal microscopy analysis of HeLa cells that were co-transfected with YFP-PARK2 (green) and mito-dsRed (red) constructs and treated with DMSO or CCCP 12 h (upper panel) and DMSO, CCCP or O/A for 2 h (lower panel). **L** and **M**, Overlap coefficiency graphs representing PARK2 (green) and mitochondria (red) colocalization during 12 h (**L**, *n* = 30) and 2 h **(M**, *n* = 30) treatments. MERGE, overlay of green and red signals. Ordinary one-way ANOVA test (**G, J** and **M**) or two-tail t-test (**F, I** and **L**) used for statistical analysis.

PARK2 migration onto mitochondria is a key rate-limiting step in the mitophagy process. To explore mitochondrial translocation of PARKIN upon different stress stimuli cytosolic and mitochondrial proteins were isolated separately by subcellular fractionation. Immunoblotting analyses and subsequent quantifications revealed increased levels of PARK2 in mitochondrial fraction following 12 h treatment with CCCP (Fig. 2A and B). In a similar manner, the amount of both ATG5-12 and PSMA7 increased in isolated mitochondria upon mitochondrial stress (Fig. 2A and B). Similarly, 2 h treatment of CCCP and O/A resulted in increased translocation of PARK2, ATG5-12 and PSMA7 to mitochondria (Fig. 2C and D) (Fig. S5, A). Moreover, confocal images revealed significant translocation of PSMA7 (Fig. 2E–G), ATG5-12 (Fig. 2H–J) and PARK2 (Fig. 2K–M) to mitochondria following long-term CCCP (12 h, 20 μM) and short term CCCP (2 h, 10 μM) or O/A (2 h, 10 μM) treatment. Collectively, these data suggest that both short-term and long-term mitochondrial stress lead to translocation of PSMA7, PARK2 and ATG5 to mitochondria.

### Stress-induced mitochondrial novel protein complexes

In order to characterize protein complexes that form during mitochondrial stress, gel filtration experiments were performed using protein extracts from PARK2 overexpressing cells. In cells that were treated with DMSO, ATG5-12, PARK2 and PARK6 protein peaks co-eluted in overlapping fractions (Fractions 2, 3, 4, MW approx. 550 kDa) in total cell lysates (Fig. S2A) and in mitochondria-free cytosolic fractions (Fig. S2E–H). In total cell lysate fractions, PARK6 was in lower amounts and corresponded to the cleaved form of the protein (55 kDa) (Fig. S2A and B). Under CCCP-induced mitochondrial stress conditions, the long, unprocessed form of the protein (63 kDa) joined the complex (Fig. S2C). On the other hand, under stress, PSMA7 protein peak shifted to separate fractions (Fractions 4, 5 and 6) (MW approx. 400 kDa) (Fig. S2A and C).

**Fig. 3:**
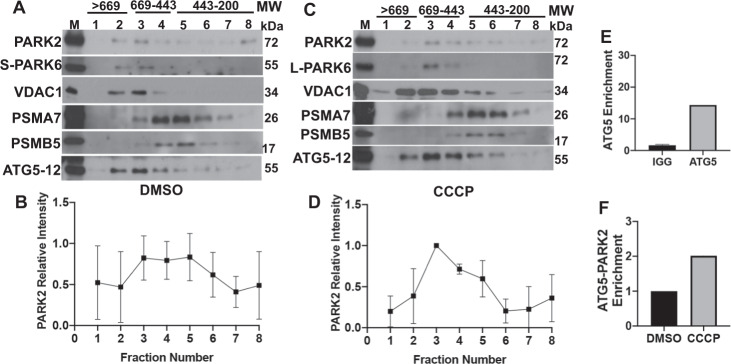
Identification of novel protein complexes that assemble under basal or mitochondrial stress conditions. **A** and **C**, Western blot images representing the levels of PARK2, PARK6, VDAC1, PSMA7, PSMB5 and ATG5-12 following gel filtration chromatography performed using mitochondrial lysates collected from YFP-PARK2 expressing HEK 293 T cells treated with either DMSO (***A****)*, or CCCP (*C)* for 12 h. M, mitochondrial extracts before fractionation. L-PARK6, uncleaved/unprocessed form of PARK6 (63 kDa). S-PARK6, cleaved/processed form of PARK6 (55 kDa). **B** and **D**, Quantifications of PARK2 relative intensity in **A** and **C** respectively (*n* = 3). *E* and *F*, Graphs showing SILAC-MS/MS enrichments of ATG5 (**E**) and PARK2-ATG5 complex (**F**) in mitochondrial fraction (*n* = 3).

In order to reveal the status of these complexes in isolated mitochondrial fractions, we purified mitochondria from PARK2 overexpressing cells, that were treated with DMSO or 10 μM CCCP for 12 h and performed gel filtration assays. Similar to the results that were obtained with total cell lysates, experiments with isolated mitochondria showed that ATG5-12, PARK2 and PARK6 eluted in the same fractions (Fractions 2, 3, 4, MW: around 550 kDa) that also contained mitochondrial outer membrane protein VDAC1. On the other hand, PSMA7 protein co-eluted with a second peak containing another 20 S proteasome subunit, PSMB5 (LPMX, MB1), in fractions 4, 5 and 6 (MW approx. 400 kDa) (Fig. 3A–D). In line with these results, proteomic analysis of the mitochondrial fraction revealed a notable increase in ATG5-PARK2 interaction following CCCP treatment (Fig. 3E and F).

Overall, gel filtration experiments and above-described protein-protein interaction tests (Y2H, Co-IP, SILAC-MS/MS and confocal microscopy analyses) established for the first time that, stress conditions resulted in the formation of dynamic higher molecular weight protein complexes on mitochondria containing autophagy and the UPS components, namely ATG5-12-PARK2-PARK6-VDAC1 complexes and PSMA7-PSMB5 and possibly other 20 S components.

### Role of proteasomes in PARK2 recruitment onto mitochondria

Given the conflicting data about the role of proteasomal activity on PARK2 recruitment [32, 37–40], we investigated whether PARK2 translocation onto mitochondria was affected by this activity. Firstly, experiments were planned with a proteasomal activity inhibitor called MG132. In order to confirm that the inhibitor was active, we showed in control experiments that MG132 affected cellular levels of bona fide targets of the proteasome, namely P27 [41] and CCND1 [42] proteins (Fig. S3A). Then, PARK2 recruitment onto mitochondria was analysed in the presence or absence of MG132 using subcellular fractionation experiments separating mitochondria from the cytosol. As previously shown, CCCP treatment resulted in the accumulation of PARK2 in mitochondrial fractions. Inhibition of proteasome activity by MG132 attenuated PARK2 mitochondrial recruitment (Fig. S3B and C). Similar results were obtained when another proteasome inhibitor 30 μM bortezomib was utilized (Fig. S3D). Also, in line with previous reports [43, 44], MG132 treatment resulted in accumulation of cleaved form (55 kDa) and reduction of the long form (63 kDa) of PARK6 (Fig. S4A and B). Also, MG132 and/or CCCP treatment did not alter the protein levels of PARK2 (Fig. S4A and C) and ATG5-12 (Fig. S4A and D) suggesting that they are not degradation targets of autophagy.

**Fig. 4:**
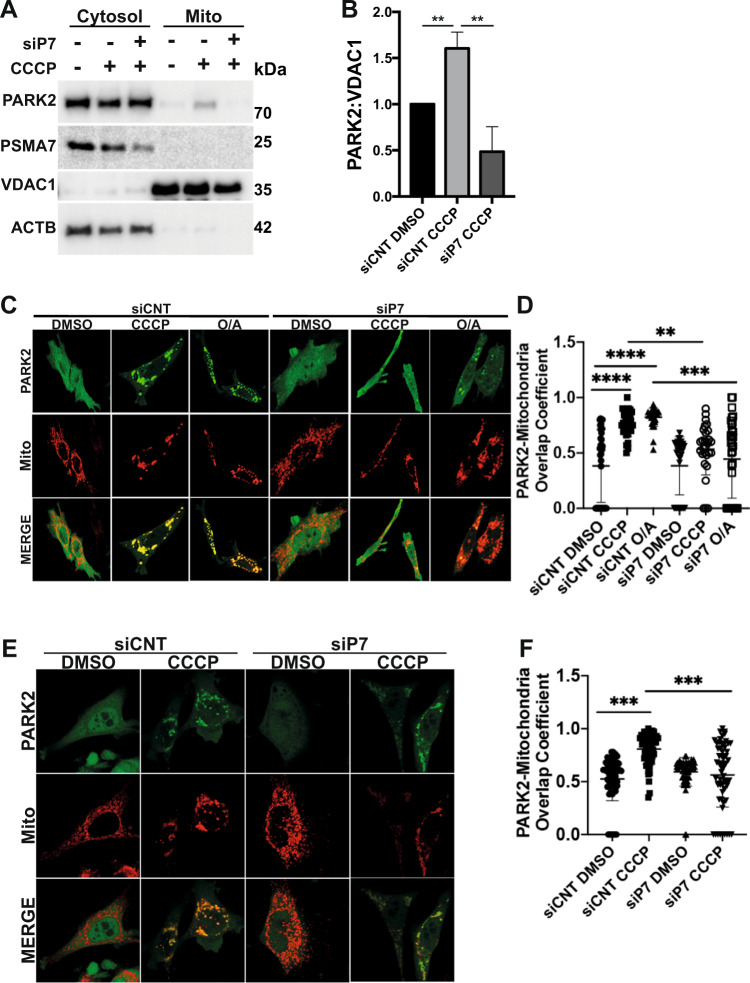
20 S proteasome subunit PSMA7 and proteasomal activity are indispensable for PARK2 recruitment to mitochondria. **A**, Representative western blot images showing PARK2, PSMA7, VDAC1 and actin beta (ACTB) protein levels in cytosolic (Cytosol) and mitochondrial (Mito) fractions of YFP-PARK2 expressing HEK 293 T cells transfected with control, non-targeting siRNAs (siCNT) or PSMA7-targeting siRNAs, (siP7) and treated with DMSO or CCCP (10 µM) for 12 h. **B** Quantification graph representing mitochondrial PARK2 levels normalized to VDAC1 from **A** (mean ± SEM, *n* = 3). **C** and **E**, Confocal microscopy analysis of YFP-PARK2 (green) and mito-dsRed (red) expressing and siCNT or siP7 transfected HeLa cells after 2 h (*C*, DMSO, 20 µM CCCP or 10 µM O/A) and 12 h (**E,** DMSO or 20 µM CCCP) of indicated treatments. MERGE, overlay of green and red signals. **D** and **F** Graphs showing quantifications of overlap coefficient of PARK2 and mitochondria (mito-dsRed) from *C* and *E* respectively (mean ± S.D., *n*≥30, one-way ANOVA).

In line with requirement of proteasome and its activity for PARK2 mitochondrial recruitment, siRNA-mediated knockdown of PSMA7 strongly decreased CCCP-stimulated accumulation of PARK2 on mitochondria (Fig. 4A and B) (Fig. S5B). We next checked intracellular localization of PARK2 protein using confocal microscopy. Under non-stimulated conditions, PARK2 localization was diffuse in the cytoplasm. CCCP- or O/A-induced short-term (2 h) (Fig. 4C and D) or CCCP-induced long-term (12 h) (Fig. 4E and F) mitochondrial stress resulted in the colocalisation of PARK2 with mitochondria in cells. When PSMA7 expression was silenced using siRNAs, PARK2 recruitment onto mitochondria was severely blocked. Quantitative analyses of confocal images confirmed that there was a significant decrease in mitochondria-localized PARK2 under these conditions (Fig. 4D and F).

**Fig. 5:**
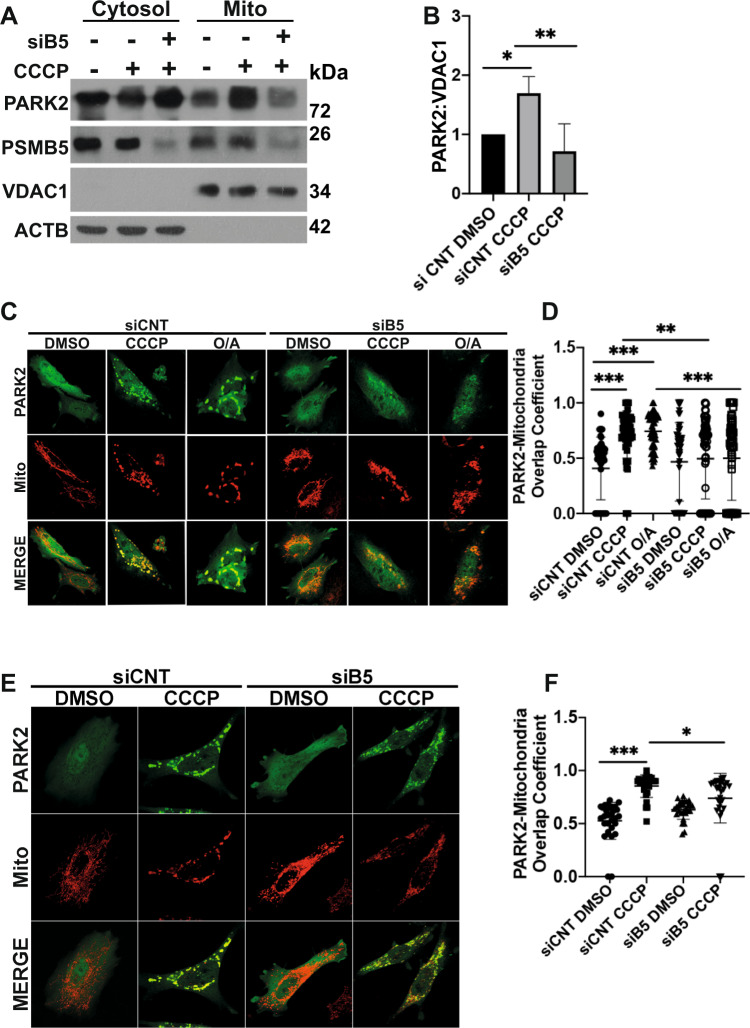
20 S proteasome subunit PSMB5 and proteasomal activity are indispensable for PARK2 recruitment to mitochondria. **A** Representative western blot images showing PARK2, PSMB5, VDAC1 and actin beta (ACTB) protein levels in cytosolic (Cytosol) and mitochondrial (Mito) fractions of YFP-PARK2 expressing HEK 293 T cells transfected with control, non-targeting siRNAs (siCNT) or PSMB5-targeting siRNAs, (siB5) and treated with DMSO or CCCP (10 µM) for 12 h. **B** Quantification graph representing mitochondrial PARK2 levels normalized to VDAC1 from **A** (mean ± SEM, *n* = 3). *C* and *E*, Confocal microscopy analysis of YFP-PARK2 (green) and mito-dsRed (red) expressing and siCNT or siB5 transfected HeLa cells after 2 h (*C*, DMSO, 20 µM CCCP or 10 µM O/A) and 12 h (*E*, DMSO or 20 µM CCCP) of indicated treatments. MERGE, overlay of green and red signals. **D** and **F** Graphs showing quantifications of overlap coefficient of PARK2 and mitochondria (mito-dsRed) from **C** and **E** respectively (mean ± S.D., *n* ≥ 30, one-way ANOVA).

To check that the observed effect of PSMA7 knockdown on PARK2 was related to its role in the 20 S proteasome, we performed similar experiments using siRNAs that are specific to another subunit of the 20 S proteasome, the PSMB5 subunit. Indeed, in gel filtration experiments, PARK2 was found in the same fractions as PSMA7 and PSMB5 (Fig. 3A and C). Similar to PSMA7, knockdown of PSMB5 hindered PARK2 recruitment onto mitochondria as assessed by subcellular fractionations (Fig. 5A and B) (Fig. S5C) and colocalization experiments (Fig. 5C–F).

**Fig. 6:**
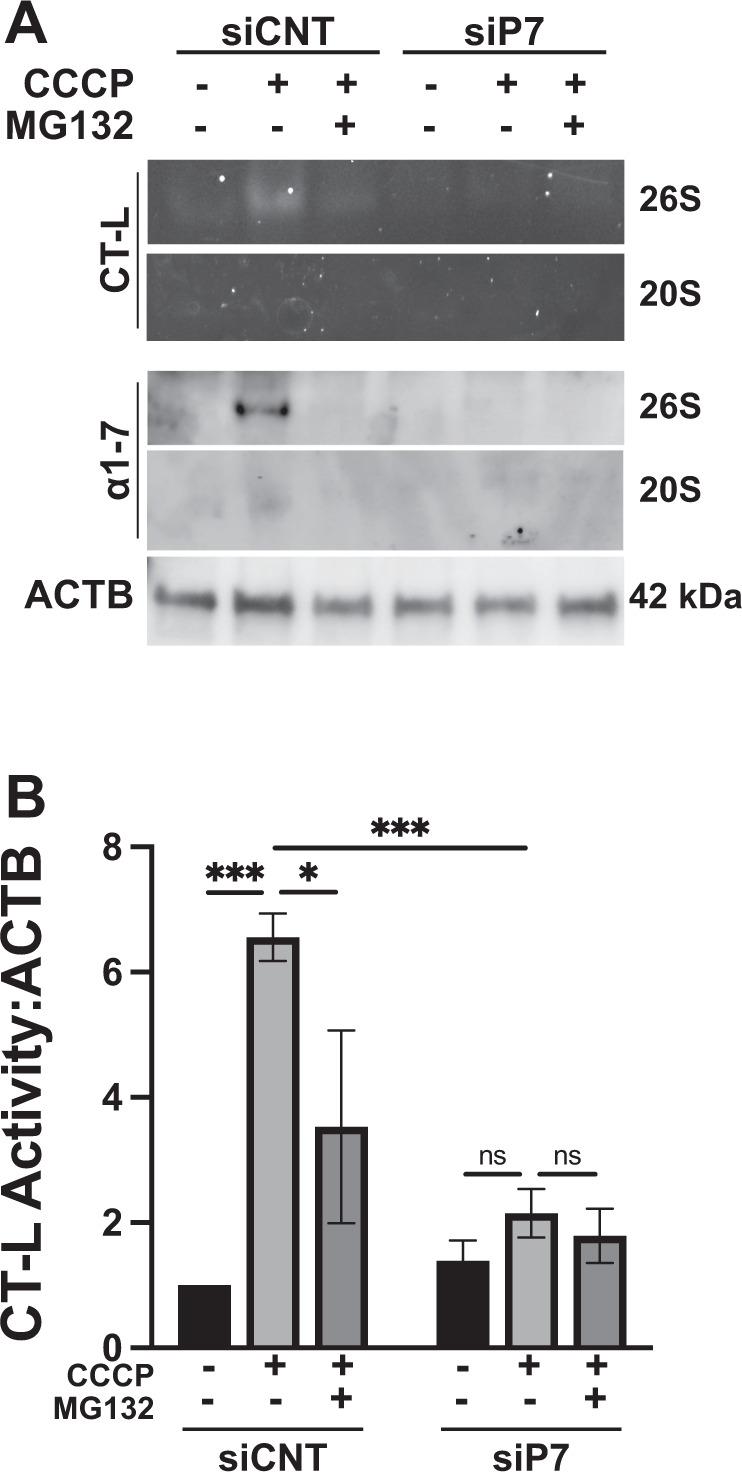
Knockdown of PSMA7 alters CCCP-induced assembly of the proteasome subunits and proteasome activity. **A** Representative native gel (upper two panels) and western blot (lower two panels) images of the 26 S and 20 S subunits of the proteasome in HA-PARK2 expressing HEK 293 T cells transfected with control siRNA (siCNT) or PSMA7 siRNA (siP7), and treated with DMSO or CCCP (20 µM) for 2 h in the absence or presence of proteasome inhibitor MG132 (2.5 h, 30 µM). Actin beta (ACTB) was used as loading control. **B** Graph representing the in-gel chymotrypsin (CT-L) proteasome activity from *A* (mean ± SEM, *n* = 3).

Furthermore, proteasome assembly and activity were analysed using in-gel proteasome activity assay under conditions during which PARK2 recruitment onto mitochondria was observed. Native gel electrophoresis was followed by in-gel proteasome activity assay, then native gels were transferred onto membranes and immunoblotting was performed using an antibody recognizing several proteasome subunits (α1−7 subunits). These experiments allowed us not only to show proteasome activity, but also revealed distinct proteasome subcomplexes (20 S and 26 S proteasomes) affected under these experimental conditions. Upon CCCP treatment, a robust increase in 26 S formation was observed (Fig. 6A), and the proteasomal activity was significantly upregulated (Fig. 6A and B). Under these conditions, knockdown of PSMA7 was sufficient to downregulate CCCP-induced proteasome activity (Fig. 6 A and B).

Collectively, we showed that: *i*, PARK2 interaction with proteasome subunits PSMA7 and PSMB5 was increased following mitochondrial stress; *ii*, under mitochondrial stress conditions, 26 S proteasome assembly and proteasomal activity was upregulated; *iii*, inhibition of proteasomal activity through knockdown of proteasome subunits as well as chemical inhibitors MG132 and bortezomib, prevented stress-induced PARK2 translocation onto mitochondria. All these results strongly suggest that PARK2 interaction with the proteasome, as well as proteasomal activity, are required for PARK2 migration onto mitochondria.

### Effect of PSMA7 Knockdown on autophagy-related functions of PARK2

**Fig. 7:**
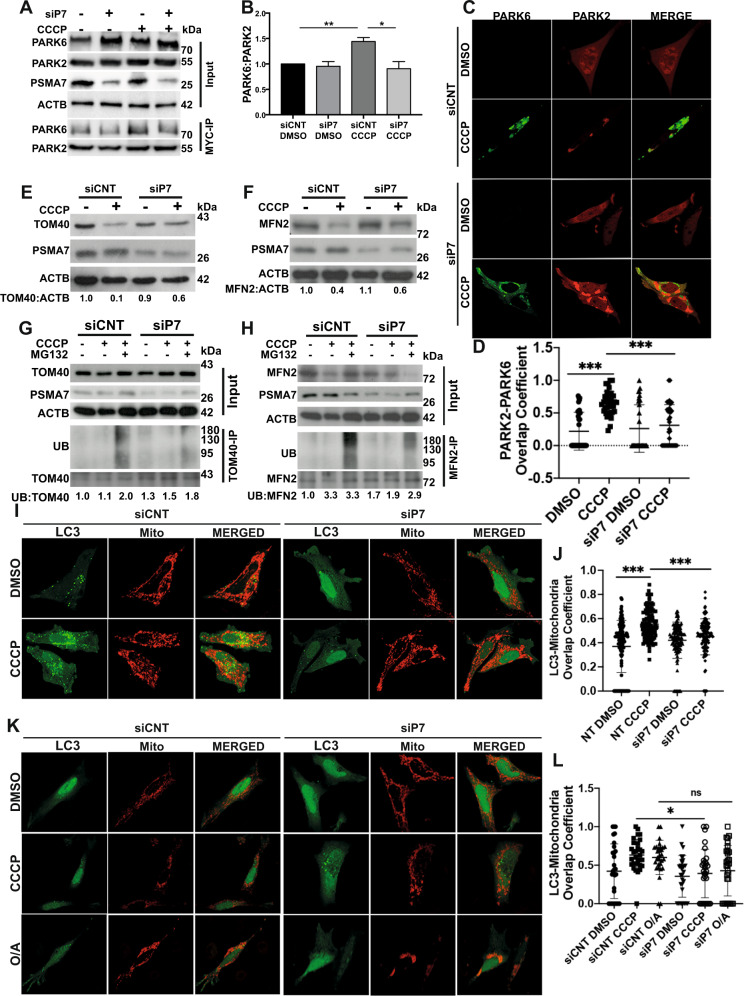
PSMA7 is required for PARK2-dependent mitophagy-related processes. **A** Representative western blot images showing PARK6, PARK2 and PSMA7 protein levels in total cell lysate (Input) and MYC-immunoprecipitated lysate (MYC-IP) of HEK 293 T cells transfected with MYC-PARK2 and GFP-PARK6 and either non-targeting control siRNA (siCNT) or PSMA7-targeting siRNA (siP7) and treated with DMSO or CCCP (10 µM) for 12 h. Actin beta (ACTB) was used as loading control. **B** Graph representing protein levels of PARK6 immunoprecipitated with MYC-PARK2 from *A* (mean ± SEM, *n* = 3). **C** Confocal microscopy analysis of GFP-PARK6 (green) and mCherry-PARK2 (red) expressing HeLa cells co-transfected with siCNT or siP7, and treated with DMSO or CCCP (10 µM) for 12 h. MERGE, overlay of green and red signals. **D** Quantification of PARK2/PARK6 overlap coefficient of microscopy analysis represented in **C** (mean ± S.D., *n* ≥ 30, one-way ANOVA). *E* and **F** Representative western blot images of TOM40 (**E**) and MFN2 (**F**) levels in MYC-PARK2 expressing HEK 293 T cells co-transfected with siCNT or siP7 and treated with either DMSO or CCCP (10 µM) for 12 h. ACTB was used as loading control. Band intensities were provided below (*n* = 3). **G** and **H** Representative western blot images of ubiquitin levels in TOM40 (**G**) and MFN2 (**H**) immunoprecipitated lysates of MYC-PARK2 expressing HEK 293 T cells co-transfected with siCNT or siP7, and treated with DMSO, CCCP (20 µM) or MG132 (30 µM) and CCCP (20 µM) for 2 h. ACTB was used as loading control. Band intensities were provided below (*n* = 3). **I** and **K,** Confocal analyses of MYC-PARK2, GFP-LC3 (green) and mito-dsRed (red) expressing HeLa cells co-transfected with siCNT or siP7, and treated with DMSO or CCCP for 12 h (*I*) and treated with DMSO, CCCP or O/A for 2 h (*K*). **J** and **L**, Quantification of overlap coeefficiency values of LC3 (green) and mitochondria (red) from *I* and *K* respectively (mean ± S.D., *n* ≥ 40, one-way ANOVA).

Next, we sought to determine functional effects of PSMA7 knockdown on autophagy-related downstream events. A significant reduction was observed in PARK2-PARK6 interaction (Fig. 7A and B) and colocalization (Fig. 7C and D) upon knockdown of PSMA7, as assessed through co-immunoprecipitation and confocal analyses, respectively. TOM40 and MFN2 have been characterized as ubiquitylation targets of PARK2 [39, 45]. Indeed, both TOM40 and MFN2 were degraded in response to CCCP-treatment; yet, PSMA7 knockdown resulted in the blockage of their degradation (Fig. 7E and F). Decreased ubiquitylation of TOM40 and MFN2 under PSMA7-depleted condition further indicated a defect in PARK2 activity (Fig. 7G and H). Furthermore, PSMA7 knockdown and subsequent impairment in PARK2 recruitment led to the inhibition of mitophagy as assessed through GFP-LC3 and mitochondria (mito-dsRed) colocalization following long term (Fig. 7I and J) or short term (Fig. 7K and L) exposure to CCCP-induced mitochondrial stress. Also, PSMA7 knockdown inhibited localization of optineurin, a selective mitophagy receptor, onto mitochondria, which further indicated that PSMA7 deficiency impaired mitophagy (Fig. S6A and B). Moreover, PSMA7 knockdown attenuated mitochondrial stress-induced decrease in mitochondrial DNA (Fig. S6C and D).

Overall, these results showed that, PSMA7, hence the proteasome is important for PARK2 recruitment and function in mitophagy.

### Role of ATG5 protein in PARK2 activation and PARK6 activity

In this study, ATG5 protein was found as another partner of the PARK2-PARK6-proteasome complex (Fig. 3 and Fig. S2). Moreover, ATG5 co-immunoprecipitated with both PARK2 and PARK6 (Fig. 1G and I; Fig. S1E and F). Therefore, in order to gain insight into the role of ATG5 in this context, we analysed the effect of ATG5 deficiency on the PARK2-PARK6 system.

**Fig. 8:**
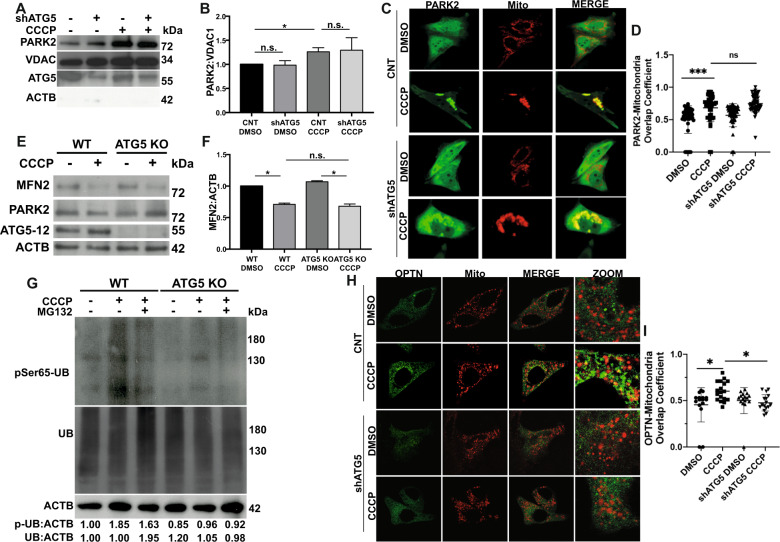
ATG5 is not required for PARK2 recruitment onto mitochondria, but is indispensable for mitophagy. **A,**Representative western blot images of PARK2, VDAC1, ATG5-12 and ACTB in mitochondrial fractions of YFP-PARK2 expressing HEK 293 T cells transfected with control vector or ATG5 specific shRNA vector (shATG5), and treated with DMSO or CCCP (10 µM) for 12 h. **B** Graph representing the quantification of mitochondria localized PARK2 from *A* (mean ± SEM, *n* = 3). **C** Representative confocal images of YFP-PARK2 (green) and mito-dsRed (red) expressing HeLa cells co-transfected with control vector (CNT) or shATG5 vector, and treated with DMSO or CCCP (10 µM) for 12 h. MERGE, overlay of green and red signals. **D** Graph showing colocalization coefficiency of PARK2 overlap with mitochondria from **C** (mean ± S.D., n≥30, one-way ANOVA). **E** Representative western blot images of MFN2, PARK2, ATG5-12 and ACTB in MYC-PARK2 over expressing wild type (WT) and ATG5 knockout (KO) HeLa cells treated with DMSO or CCCP (10 µM) for 12 h. ACTB was used as loading control. **F**, Quantification of MFN2 levels from **E** (mean ± SEM, *n* = 3). **G**, Representative western blot images of phosphoSer65-ubiquitin (pSer65-UB) and total ubiquitin (UB) levels in MYC-PARK2 expressing wild type (WT) and ATG5 knockout (KO) HeLa cells treated with DMSO, CCCP (20 μM, 2 h) or MG132 (30 μM, 2.5 h) and CCCP (20 μM, 2.5 h). Band intensities were marked below (*n* = 3). **H** Confocal images of GFP-optineurin (OPTN, green) and mito-dsRed (red) expressing HeLa cells co-transfected with control vector or shATG5 vector, and treated with DMSO or CCCP (10 μM) for 12 h. MERGE, overlay of green and red signals. ZOOM, zoomed images of particular area. **I** Quantification graph showing the overlap coefficiency values of optineurin dots (green) and mitochondria (red) from *H* (mean ± S.D., *n* ≥ 30, one-way ANOVA).

We next investigated whether PARK2 recruitment onto mitochondria was ATG5-dependent. We observed that the amount of PARK2 in the mitochondrial fraction of cells was not altered upon knockdown of ATG5 (Fig. 8A and B) (Fig. S5, D). Moreover, PARK2 colocalization with mitochondria in CCCP-treated cells was not affected in shATG5 transfected cells (Fig. 8C and D). Mitochondrial levels of VDAC1 protein did not respond to CCCP treatment, even though it is a reported ubiquitylation target of PARK2 [46]. VDAC1 levels were also comparable in ATG5-deficient cells (Fig. 8A). On the other hand, levels of another PARK2 target, MFN2, decreased in a similar manner in the absence or presence of ATG5 (Fig. 8E and F). Collectively, these results suggest that loss of ATG5 does not alter PARK2 recruitment onto mitochondria and the levels of those ubiquitylation substrates that are degraded.

Previous studies showed that PARK2 as well as the ubiquitin protein itself are phosphorylated by PARK6 on their respective Ser65 residues, creating a feed-forward loop of PARK2 activation by PARK6 upon mitochondrial stress. Unfortunately, due to antibody-related problems, we could not obtain conclusive results about PARK2 phosphorylation by PARK6. Hence, we tested ubiquitin phosphorylation at Ser65 by PARK6 under these conditions. In control cells, CCCP treatment led to a notable increase in MG132-sensitive ubiquitin and phospho-ubiquitin (pSer65-UB, p-UB) levels (Fig. 8G). On the other hand, in ATG5 deficient cells, CCCP-induced and MG132-sensitive ubiquitin and phosho-ubiquitin increase was markedly attenuated (Fig. 8G). Hence, although ATG5 deficiency did not affect PARK2 recruitment and degradation of some of its substrates, a general reduction in ubiquitylation and especially ubiquitin phosphorylation, was observed.

Optineurin was shown by several groups to bind ubiquitylated, and especially phospho-ubiquitylated proteins on mitochondria and bridge between these proteins and autophagy components such as LC3. In line with the reduction observed in ubiquitin/phospho-ubiquitin levels upon loss of ATG5, Optineurin that has accumulated on depolarized mitochondria was significantly reduced upon loss of ATG5 (Fig. 8H and I).

All these data indicate that ATG5, a component of novel higher molecular weight mitochondrial complexes that are described in this study, is important for PARK2 and PARK6 activity and regulation of upstream stages of the mitophagy process.

## Discussion

In this study, we showed that: *i*, direct protein-protein interactions exist between autophagy proteins and UPS components; *ii*, previously unknown protein complexes of ATG5-12, 20 S proteasome components, including PSMA7, PARK2 and PARK6 were discovered and they migrated to mitochondria under stress conditions; *iii*, PSMA7, PSMB5 and the proteasomal activity were required for PARK2 recruitment onto mitochondria, PARK2 activation and its mitophagy-related functions; *iv*, ATG5-12, PARK2 and PARK6 were found to form a separate complex; *v*, ATG5 was required for proper PARK2 and PARK6 activity. Although PARK2 could still accumulate on mitochondria in ATG5-depleted cells, ubiquitylation and ubiquitin phosphorylation defects were observed; *vi*, under these conditions, recruitment of the selective mitophagy receptor optineurin and mitophagy was blocked.

There are indications about connections between autophagy and UPS and their possible co-regulation [12, 31]. Using yeast-two-hybrid, co-immunoprecipitation, confocal microscopy, SILAC-MS/MS and gel filtration analyses, we demonstrated that ATG5-12, 20 S proteasome components PSMA7 and PSMB5, and mitophagy proteins PARK2 and PARK6 interacted with each other and formed high molecular weight protein complexes. We observed that these complexes are highly dynamic and they respond to mitophagy-inducing signals. Gel filtration experiments using mitochondrial fractions showed that a major 550 kDa complex containing ATG5-12, PARK2, uncleaved PARK6 and VDAC1 formed following CCCP treatment.

Moreover, 20 S proteasome components were found to be in a dynamic interaction with this complex. The approximate molecular weight of the 20 S proteasome is 750 kDa [47]. It is possible that under our experimental conditions (e.g., RIPA buffer), 20 S proteasome dissociated from the complex, and only strongly and directly interacting components PSMA7 and PSMB5 could be identified as interaction partners.

So far, the role of the proteasomal activity on PARK2 mitochondrial recruitment is not clear [32, 37–40]. In our system, mitochondrial stress promoted 26 S proteasome assembly and resulted in a robust increase in its activity. Under these conditions, inhibition of proteasomal activity by drugs MG132 or bortezomib, or using genetic knockdown of proteasome components PSMA7 and PSMB5 blocked PARK2 recruitment onto mitochondria. Targets that were affected by proteasome inhibition in this scenario are currently unknown. In a previous study, a number of ubiquitylated proteins were reported to accumulate upon MG132 treatment and in a PARK2-independent manner [48]. Recruitment of PARK2 onto mitochondria might depend on the degradation of a subset of these proteins by the UPS system.

Previous studies showed that phosphorylation of ubiquitin and PARK2 itself by PARK6 resulted in the decoration of mitochondria with proteins tagged with phospho-ubiquitin and created a feed-forward loop leading to further PARK2 recruitment and activation [43, 49]. We showed that loss of ATG5 decreased general ubiquitylation to a certain extent, but its prominent effect was on phospho-ubiquitin levels. We did not observe a defect in the degradation of proteins regulating mitochondrial fusion and transport (e.g., MFN2 and TOM40) in ATG5-deficient cells. This can be explained by the role of ATG5 as a regulator of ubiquitin phosphorylation on more stable mitochondrial proteins that might serve as docking targets for mitophagy receptors, including optineurin. For example, previous studies showed that, VDAC1 was ubiquitylated in a PARK2-dependent manner, and VDAC1 proteins were required for mitophagy [19, 50]. Although it should be ubiquitylated in our system, we did not observe a notable reduction in the levels of VDAC1 protein in ATG5-deficient cells. Hence in the 550 kDa complex, ATG5 might function in the fine-tuning of PARK2-PARK6-dependent ubiquitin phosphorylation resulting in priming of proteins, such as VDAC1. This might facilitate subsequent mitophagy stages involving autophagy receptor binding and autophagic machinery build-up on mitochondria. In line with this, we observed a defect in optineurin recruitment onto mitochondria in ATG5-deficient cells. Binding of mitophagy receptors to ubiquitylated mitochondrial proteins is indeed crucial for the assembly of the autophagy machinery components, such as ULK1, DFCP1 (ZFYVE1) and WIPI1 and progression of mitophagy [51].

PARK2-dependent mechanisms are functional in neurons and in some other cell types [52–54]. Moreover, introduction of PARK2 into PARK2-deficient cell types result in overriding of the system towards PARK2-dependent mitophagy [55, 56]. On the other hand, PARK2-independent mechanisms also exist. For instance, AMBRA1 was shown to facilitate recruitment of autophagosomes decorated with ubiquitin and LC3 in a PARK2-independent manner, suggesting involvement of other mitophagy-specific E3 ligases during mitochondrial stress [57–59]. Furthermore, ubiquitylation-independent mechanisms, such as FUNDC1-dependent mitophagy was described [27, 60]. Whether ATG5 plays a similar role in PARK2-independent mitophagy mechanisms needs to be clarified by further studies.

Altogether, our study revealed an intricate interaction between autophagy machinery components, PARK2/PARK6 E3 ubiquitin ligase system, 20 S proteasome subunits and proteasomal activity during mitophagy, and introduced ATG5-12 as a co-regulator of both autophagy and the UPS.

## Materials and methods

### Yeast two-hybrid screening

Yeast two-hybrid screens were described elsewhere [35]. Briefly, a full-length human Atg5 cDNA in the pGBKT7 bait vector (CLONTECH) was screened against a thymus cDNA library (CLONTECH) in the AH109 yeast strain. Colonies that are formed under selection conditions (lack histidine, adenine, tryptophan and leucin) were analysed by sequencing. Clone 47 corresponded to a sequence coding for PSMA7 amino acids 174-248.

### Cell culture and transfection

Human embryonic kidney HEK 293 T and HeLa cells were cultured in DMEM (Dulbecco’s modified Eagle’s medium; PAN, P04-03500) supplemented with 10% (v/v) fetal bovine serum (FBS; PAN, P30-3302) and antibiotics (100 units/mL penicillin and 100 μg/mL streptomycin; Biological Industries, BI03-031-1B) and 1% L-glutamine (Biological Industries, BI03-020-1B) in a 5% CO_2_ humidified incubator. Cells were tested negative for mycoplasma. Transient transfections of HEK 293 T and HeLa cells were achieved using the calcium-phosphate precipitation method according to standart protocols [61]. SiRNA mediated knockdown experiments performed by using non-targeting siRNA (siCNT) (Dharmacon SiGENOME, D- 001210-01-20) or PSMA7 targeting siRNA (siP7) (Dharmacon SiGENOME Smart Pool SiRNA PSMA7, M- 004209-00-0010) purchased from Thermo Scientific. For generation of *ATG5* KO HeLa cells, HEK 293 T cells were transfected with either control or *ATG5* targeting gRNA containing lentiCRISPR v2 along with psPAX2 (Addgene, 12260) and pMD2.G (Addgene, 12259) as previously described [62]. HeLa cells were transduced with lentiviral particles collected from media of HEK 293 T cells and monoclones were selected using puromycin.

To induce mitochondrial depolarization, cells were incubated with CCCP (Sigma-Aldrich, C2759) for either 2 h (10 μM) or 12 h (10 μM), O/A (Oligomycin Sigma-Aldrich O4876; Antimycin A Sigma-Aldrich, A8674) for 2 h (10 μM) and STAURO (Sigma-Aldrich, S5921) for 12 h (1 μM). Proteasome inhibitors MG132 (Sigma-Aldrich, M8699) or bortezomib (Sigma-Aldrich, S1013) was added 30 min prior to the addition of mitochondrial uncoupler.

### Plasmids and constructs

pMXs-IP HA-PARK2 (38248), YFP-PARK2 (23955), pRK5-MYC-PARK2 (17612), mCherry-PARK2 (23956), pCDNA-DEST47 PINK1-C-GFP (13316), optineurin(OPTN)-EGFP (27052) and pmCherry-ATG5 (13095) plasmids were purchased from Addgene. FLAG-human ATG5 (RC235557), non-tagged human ATG5 (SC128244) and MYC-DDK-PSMA7 (RC201169) were purchased from Origene. GFP-LC3 construct also described [63]. GFP-PSMA7 vector was created by cloning using the MYC-DDK-PSMA7 construct into PEGFP-N3-empty vector. ATG5 KO lentiCRISPR V2 vector was created by cloning ATG5 targeting gRNA into lentiCRISPR V2 (Addgene, 52961) using BSMBI.

### Protein extraction and western blotting

HEK 293 T, HeLa and MEF cell pellets were dissolved in RIPA buffer (1 M Tris-HCl, pH 7.6, 5% deoxycholic acid (Sigma-Aldrich, 30970), 10% NP-40 (Sigma-Aldrich, 74385), and 0.5 M NaCl (Applichem, A2942) supplemented with protease inhibitiors (Roche, 04-693-131-001) and 1 mM phenylmethylsulfonyl fluoride (PMSF; Sigma-Aldrich, P7626). Following centrifugation at 16800 x g for 15 min and protein concentrations were determined using the Bradford assay as per manufacturer’s instructions (Sigma-Aldrich, B6916). Proteins were loaded into the 12%-15% SDS-polyacrylamide gels and transferred onto nitrocellulose membranes. Following blockage with 5% (w/v) non-fat dried milk (Applichem, A0830) or BSA (Sigma-Aldrich, A3059) in 1xPBST (PBS and 0.05% Tween 20, pH 7.4), membranes were incubated with primary, and after washes, with secondary antibodies. Immunoreactive bands were visualised using either X-ray films (Mediphot X-O/RP) or ChemiDoc MP Imaging systems (Bio-Rad). Band intensities were quantified using either ImageJ or ImageLab (Bio-Rad) software. Original western blots were provided as supplementary material.

Following primary antibodies were used: Anti-PARK2 ab 1:2000 (Santa Cruz Biotech., sc-32282), anti-TOM40 ab 1:1000 (Santa Cruz Biotech., sc-11414), anti-Flag 1:4000 (Sigma, F3290), anti- ATG5 N-terminal ab 1:2000 (Sigma, AO856), Anti-LC3 1:1000 (Novus, 2331), Anti-MFN2 1:1000 (Sigma, M6444), Anti-Actin (ACTB) 1:10000 (Sigma, A5441), Anti- PSMA7 1:1000 (Enzo Lifesciences, PW8120), Anti-PSMB5 1:1000 (Enzo Lifesciences, PW8895), Anti-VDAC1 1:1000 (Millipore, AB10527), Anti- Tim23 1:1000 (BD Transduction, 611222), Anti-Pink1 1:1000 (Novus, BC100-494), Anti-Ubiquitin ab (P4D1) 1:1000 (Santa Cruz Biotech., Sc-8017), Anti-pSer65 Ubiquitin ab 1:1000 (Millipore, ABS1513) Anti-Proteasome 20 S α1-7 (Enzo Lifesciences, PW8155). Secondary mouse or rabbit antibodies coupled to horseradish peroxidase were used: (Jackson Immunoresearch Laboratories, ant-mouse-HRP 115035003 and anti-rabbit-HRP 111035144, dilutions 1:10.000).

### Immunoprecipitation

Immunoprecipitation experiments were performed as previously described [61]. In brief, 2×10^6^ cells were harvested 48 or 72 h after transfection and washed with ice-cold 1×PBS. Following protein lysis and determination of protein concentration, 0.5-2 mg protein extract was loaded onto either A (Protein A-Agarose: sc-2001) or G (Protein G PLUS-Agarose sc-2002) agarose beads that had already been hybridized with respective antibodies for 4-6 h at 4 ^°^C cold room. Beads loaded with protein extracts were incubated o/n at 4 °C overnight on a rotator. Following thorough washing by centrifugation, equal amount of immunoprecipitated and input (proteins only) proteins were loaded into bis-acrylamide gel. Gel electrophoresis and immunoblotting were performed as described in the section above.

### Subcellular fractionation

30 × 10^7^ cells were harvested and washed 3 times in ice-cold 1xPBS. Cell pellets were resuspended in homogenization buffer (600 mM sucrose (Sigma-Adrich, S9378), 10 mM Tris-HCl (Trisma Base; Sigma-Aldrich, T1503), pH 7.4 supplemented with 1 mM EDTA (Calbiochem, 324503) pH 8.0, 0.1% (v/v) protease inhibitor cocktail (Sigma-Aldrich, P8340), 1 mM NaF (Fluka, 71527), 0.2 mM NaVO_3_ (Sigma-Aldrich, 450243) and 0.1 mM PMSF). Following centrifugation at 500 x g at 4 °C, pellets were resuspended in 1 mL homogenization buffer and homogenized in a glass potter (25 strokes). Extracts were centrifuged at 1500 x g and then at 3000 × g at 4 °C. Crude mitochondria were separated following centrifugation at 12000 × g for 15 min, and then resuspended in the isolation buffer (250 mM sucrose, 10 mM MOPS (Calbiochem, 475898) pH 7.2, 1 mM EDTA pH 8.0, 0.1% (v/v) Protease Inhibitor Cocktail (Sigma-Aldrich, P8340), 1 mM NaF, 0.2 mM NaVO_3_ and 0.1 mM PMSF) and added on top of the pre-cooled sucrose density gradient consisting of four distinct sucrose layers containing 60, 32, 23 or 15% sucrose. After ultracentrifugation (Beckman Coulter, Optima Max-XP) at 134000 × g at 4 °C for 1 h, mitochondria were observed in between sucrose layers 60-32%. After washes in isolation buffer, pellets were resuspended in the RIPA buffer. Equal amount of mitochondrial protein lysate was resolved in bis-acrylamide gels following estimation of protein concentration using Bradford reagent as previously described.

### Gel filtration

For separation of protein complexes, a Superdex 200 10/300GL (separation range 10 to 600 kDa, GE Healthcare, 17-5175-02) or a SuperoseTM 6 10/300 GL (separation range 5 to 5000 kDa, GE Healthcare, 17-5172-01) columns were used. Sigma molecular weight kit was used for the calibration of the system (Sigma, MWGF-1000). Chromatography analyses were performed using an AKTA Prime FPLC system (AKTA FPLC UPC900 / P920 System / Frac 900 fraction collector, GE Amersham Pharmacia, US). For separation of proteins, a modification of a previously reported protocol was used [4, 35]. Briefly, chromatography column was calibrated using a degassed balancing buffer (1:1, 0.05% glycerol PBS: RIPA buffer) and optimize flux, absorbance and pressure parameters were optimized (Pressure: 1.5 MPa; Flux velocity: 0.5 ml/min.; Fraction volume: 0.5 ml; Sample Loop volume: 500 μl.). 5- 7.5 mg protein extracts from cells were diluted in 500 μl RIPA buffer, and loaded onto coulumns and ran at 4 °C. 500 µl fractions were collected using a fraction collector. Columns were then washed with 108 ml deionized water (3x column bed volume), and re-calibrated with the balancing buffer. Immunoblotting of the collected fractions were performed as described.

### Native gel and in-gel proteasome activity assay

Native gel electrophoresis and in-gel proteasome activity were performed as previously described [64]. Briefly, HEK 293 T cells were harvested and lysed using TSDG lysis buffer. Briefly, protein concentration was determined as described above. Equal amount of proteins were loaded into 10% SDS-polyacrylamide gel. Gel was run the gel at 150 V for 4 h at 4 °C cold room. To assess in-gel proteasome activity, native gel was incubated in solution containing Suc-LLVY-AMC (BML-P802) for 30 min at 37 °C. In-gel Proteasome chymotrypsin-like (CT-L) activity was measured at an excitation wavelength of 380 nm and emission wavelength of 460 nm. Immunoblotting was performed as described above. CT-L activity and immunoreactive band intensities were calculated using ImageLab software (Bio-Rad).

### SILAC labelling and LC-MS/MS

SILAC-based mass spectrometry analysis, LC-MS/MS and MS data analysis were performed as described [35, 65]. Tri-SILAC labeled FLAG-ATG5 expressing HEK 293 T cell extracts and mitochondria that were isolated from them were used in the analyses.

### Immunofluorescence analysis and quantification of relative fluorescence intensity or colocalization coefficient

Immunofluorescence-based microscopy analyses were performed as previously described [61, 63]. Briefly, HeLa and HEK 293 T cells were seeded onto poly-L-lysine coated cover slides. Following 48 h post-transfection and indicated treatments, cells were fixed with ice-cold 4% paraformaldehyde (PFA, pH 7.4). Cells were then permeabilized using 0,1% saponin in BSA solution. Microscopy analyses was performed by 63×/1.4 oil immersion DIC Plan Apo objective with confocal microscopes (LSM710; Carl Zeiss, Inc., Germany or Leica Microsystems, DMI8 SP8 DLS/CS). Relative fluorescence intensities of mitotracker RED were analyzed using Histogram tool from ZEN software (LSM710; Carl Zeiss, Inc., Germany). Nuclear area was excluded using drawing tool. Relative fluorescence intensity in each experimental set up was normalized based on the mean value of DMSO or siCNT DMSO conditions. Colocalization coefficient of mitochondria with LC3, PSMA7, ATG5-12, PARK6 or PARK2 was analyzed using colocalization tool from Zen software (LSM710; Carl Zeiss, Inc., Germany) and then ordinary one-way ANOVA statistical analysis were performed using Prism 8 software for interpretation of the data.

### Digital image processing

Fluorescence and western blot images were acquired as uncompressed bitmapped digital data (TIFF format) and processed using Adobe Photoshop CC2019, version 20.0.1.

### Statistical analyses

Statistical analyses were performed by using Student’s t-test unless otherwise stated. Data were represented as means S.E.M. or S.D. of at least 3 independent experiments (*n* ≥ 3) unless otherwise stated [66]. *p* values ≤ 0.05 are represented as *, ≤ 0.01 as **, ≤ 0.001 as ***, and n.s. (not significant) if > 0.05.

### Reporting summary

Further information on research design is available in the Nature Research Reporting Summary linked to this article.

## Supplementary information

Supplementary Figure Legends

Figure S1

Figure S2

Figure S3

Figure S4

Figure S5

Figure S6

Figure S7

Figure S8

Figure S9

Figure S10

Figure S11

Figure S12

Figure S13

Figure S14

Figure S15

Reporting Summary

## Data Availability

Data required to evaluate the conclusions of this study are presented in the paper and/or the Supplementary Materials. SILAC-MS/MS-based interactome data are available from the corresponding author upon direct request by e-mail.

## References

[1] Mizushima N (2007). Autophagy: Process and function. Genes Dev.

[2] Chen Y, Klionsky DJ (2011). The regulation of autophagy - Unanswered questions. J Cell Sci.

[3] Walker S, Chandra P, Manifava M, Axe E, Ktistakis NT (2008). Making autophagosomes: Localized synthesis of phosphatidylinositol 3-phosphate holds the clue. Autophagy.

[4] Mizushima N, Kuma A, Kobayashi Y, Yamamoto A, Matsubae M, Takao T (2003). Mouse Apg16L, a novel WD-repeat protein, targets to the autophagic isolation membrane with the Apg12-Apg5 conjugate. J Cell Sci.

[5] Mizushima N, Komatsu M (2011). Autophagy: Renovation of cells and tissues. Cell.

[6] Hanada T, Noda NN, Satomi Y, Ichimura Y, Fujioka Y, Takao T (2007). The Atg12-Atg5 conjugate has a novel E3-like activity for protein lipidation in autophagy. J Biol Chem.

[7] Ohsumi Y, Mizushima N (2004). Two ubiquitin-like conjugation systems essential for autophagy. Semin Cell Dev Biol.

[8] Mizushima N, Levine B (2010). Autophagy in mammalian development and differentiation. Nat Cell Biol.

[9] Scott SV, Guan J, Hutchins MU, Kim J, Klionsky DJ (2001). Cvt19 is a receptor for the cytoplasm-to-vacuole targeting pathway. Mol Cell.

[10] Gatica D, Lahiri V, Klionsky DJ (2018). Cargo recognition and degradation by selective autophagy. Nat Cell Biol.

[11] Palikaras K, Lionaki E, Tavernarakis N (2018). Mechanisms of mitophagy in cellular homeostasis, physiology and pathology. Nat Cell Biol.

[12] Kocaturk NM, Gozuacik D. Crosstalk between mammalian autophagy and the ubiquitin-proteasome system. Front. Cell Dev. Biol. 2018 6:128. 10.3389/fcell.2018.00128 PMC6175981 30333975

[13] Heinemeyer W, Ramos PC, Dohmen RJ (2004). The ultimate nanoscale mincer: Assembly, structure and active sites of the 20S proteasome core. Cell Mol Life Sci.

[14] Groll M, Huber R (2003). Substrate access and processing by the 20S proteasome core particle. Int J Biochem Cell Biol.

[15] Hu XT, Chen W, Wang D, Shi QL, Zhang FB, Liao YQ (2008). The proteasome subunit PSMA7 located on the 20q13 amplicon is overexpressed and associated with liver metastasis in colorectal cancer. Oncol Rep..

[16] Xia S, Tang Q, Wang X, Zhang L, Jia L, Wu D (2019). Overexpression of PSMA7 predicts poor prognosis in patients with gastric cancer. Oncol Lett.

[17] Sugimoto K, Hiwasa T, Shibuya K, Hirano S, Beppu M, Isose S (2018). Novel autoantibodies against the proteasome subunit PSMA7 in amyotrophic lateral sclerosis. J Neuroimmunol.

[18] Yamano K, Youle RJ (2013). PINK1 is degraded through the N-end rule pathway. Autophagy.

[19] Sun Y, Vashisht AA, Tchieu J, Wohlschlegel JA, Dreier L (2012). Voltage-dependent anion channels (VDACs) recruit parkin to defective mitochondria to promote mitochondrial autophagy. J Biol Chem.

[20] Kazlauskaite A, Kondapalli C, Gourlay R, Campbell DG, Ritorto MS, Hofmann K (2014). Accelerated publication: Parkin is activated by PINK1-dependent phosphorylation of ubiquitin at Ser65. Biochem J.

[21] Kondapalli C, Kazlauskaite A, Zhang N, Woodroof HI, Campbell DG, Gourlay R (2012). PINK1 is activated by mitochondrial membrane potential depolarization and stimulates Parkin E3 ligase activity by phosphorylating Serine 65. Open Biol.

[22] Kane LA, Lazarou M, Fogel AI, Li Y, Yamano K, Sarraf SA (2014). PINK1 phosphorylates ubiquitin to activate parkin E3 ubiquitin ligase activity. J Cell Biol.

[23] Schweers RL, Zhang J, Randall MS, Loyd MR, Li W, Dorsey FC (2007). NIX is required for programmed mitochondrial clearance during reticulocyte maturation. Proc Natl Acad Sci USA.

[24] Sandoval H, Thiagarajan P, Dasgupta SK, Schumacher A, Prchal JT, Chen M (2008). Essential role for Nix in autophagic maturation of erythroid cells. Nature.

[25] Ding WX, Ni HM, Li M, Liao Y, Chen X, Stolz DB (2010). Nix is critical to two distinct phases of mitophagy, reactive oxygen species-mediated autophagy induction and Parkin-ubiquitin-p62-mediated mitochondrial priming. J Biol Chem.

[26] Hsieh CH, Shaltouki A, Gonzalez AE, Bettencourt da Cruz A, Burbulla LF, St Lawrence E (2016). Functional Impairment in Miro Degradation and Mitophagy Is a Shared Feature in Familial and Sporadic Parkinson’s Disease. Cell Stem Cell.

[27] Chen M, Chen Z, Wang Y, Tan Z, Zhu C, Li Y (2016). Mitophagy receptor FUNDC1 regulates mitochondrial dynamics and mitophagy. Autophagy.

[28] Wei Y, Chiang WC, Sumpter R, Mishra P, Levine B (2017). Prohibitin 2 Is an Inner Mitochondrial Membrane Mitophagy Receptor. Cell.

[29] Chakraborty J, Stockum S, Marchesan E, Caicci F, Ferrari V, Rakovic A et al. USP 14 inhibition corrects an in vivo model of impaired mitophagy. EMBO Mol Med. 2018;10:9014. 10.15252/emmm.201809014 PMC6220287 30249595

[30] Korolchuk VI, Menzies FM, Rubinsztein DC (2010). Mechanisms of cross-talk between the ubiquitin-proteasome and autophagy-lysosome systems. FEBS Lett.

[31] Korolchuk VI, Mansilla A, Menzies FM, Rubinsztein DC (2009). Autophagy Inhibition Compromises Degradation of Ubiquitin-Proteasome Pathway Substrates. Mol Cell.

[32] Tanaka A, Cleland MM, Xu S, Narendra DP, Suen DF, Karbowski M (2010). Proteasome and p97 mediate mitophagy and degradation of mitofusins induced by Parkin. J Cell Biol.

[33] Bragoszewski P, Turek M, Chacinska A. Control of mitochondrial biogenesis and function by the ubiquitin - Proteasome system. Open Biol. 2017;7:7. 10.1098/rsob.170007 PMC5413908 28446709

[34] Cohen-Kaplan V, Livneh I, Avni N, Fabre B, Ziv T, Kwon YT (2016). p62- and ubiquitin-dependent stress-induced autophagy of the mammalian 26S proteasome. Proc Natl Acad Sci USA.

[35] Erbil S, Oral O, Mitou G, Kig C, Durmaz-Timucin E, Guven-Maiorov E (2016). RACK1 is an interaction partner of ATG5 and a novel regulator of autophagy. J Biol Chem.

[36] Dächsel JC, Lücking CB, Deeg S, Schultz E, Lalowski M, Casademunt E (2005). Parkin interacts with the proteasome subunit α4. FEBS Lett.

[37] Chan NC, Salazar AM, Pham AH, Sweredoski MJ, Kolawa NJ, Graham RLJ (2011). Broad activation of the ubiquitin-proteasome system by Parkin is critical for mitophagy. Hum Mol Genet.

[38] Okatsu K, Koyano F, Kimura M, Kosako H, Saeki Y, Tanaka K (2015). Phosphorylated ubiquitin chain is the genuine Parkin receptor. J Cell Biol.

[39] Yoshii SR, Kishi C, Ishihara N, Mizushima N (2011). Parkin mediates proteasome-dependent protein degradation and rupture of the outer mitochondrial membrane. J Biol Chem.

[40] Wang H, Song P, Du L, Tian W, Yue W, Liu M (2011). Parkin ubiquitinates Drp1 for proteasome-dependent degradation: Implication of dysregulated mitochondrial dynamics in Parkinson disease. J Biol Chem.

[41] Boulanger J, Vézina A, Mongrain S, Boudreau F, Perreault N, Auclair BA (2005). Cdk2-dependent Phosphorylation of Homeobox Transcription Factor CDX2 Regulates Its Nuclear Translocation and Proteasome-mediated Degradation in Human Intestinal Epithelial Cells. J Biol Chem.

[42] Gennaro VJ, Stanek TJ, Peck AR, Sun Y, Wang F, Qie S (2018). Control of CCND1 ubiquitylation by the catalytic SAGA subunit USP22 is essential for cell cycle progression through G1 in cancer cells. Proc Natl Acad Sci.

[43] Narendra DP, Jin SM, Tanaka A, Suen DF, Gautier CA, Shen J et al. PINK1 is selectively stabilized on impaired mitochondria to activate Parkin. PLoS Biol 2010;8:1000298. 10.1371/journal.pbio.1000298 PMC2811155 20126261

[44] Kazlauskaite A, Kondapalli C, Gourlay R, Campbell DG, Ritorto MS, Hofmann K et al. Parkin is activated by PINK1-dependent phosphorylation of ubiquitin at Ser ^65^. Biochem J. 2014. 10.1042/BJ20140334 PMC4000136 24660806

[45] Gegg ME, Cooper JM, Chau KY, Rojo M, Schapira AH V, Taanman JW. Mitofusin 1 and mitofusin 2 are ubiquitinated in a PINK1/parkin-dependent manner upon induction of mitophagy. Hum Mol Genet 2010;19:ddq419. 10.1093/hmg/ddq419 PMC3583518 20871098

[46] Geisler S, Holmström KM, Skujat D, Fiesel FC, Rothfuss OC, Kahle PJ et al. PINK1/Parkin-mediated mitophagy is dependent on VDAC1 and p62/SQSTM1. Nat Cell Biol 2010;12:ncb2012. 10.1038/ncb2012 20098416

[47] Tanaka K, Tamura T, Tanahashi N, Tsurumi C. Protein and gene structures of 20S and 26S proteasomes. Adv. Exp. Med. Biol. 1996:18795. 10.1007/978-1-4613-0335-0_23 8861010

[48] Sulkshane P, Duek I, Ram J, Thakur A, Reis N, Ziv T et al. Inhibition of proteasome reveals basal mitochondrial ubiquitination. J Proteomics. 2020;229:103949. 10.1016/j.jprot.2020.103949 32882436

[49] Narendra D, Tanaka A, Suen DF, Youle RJ (2008). Parkin is recruited selectively to impaired mitochondria and promotes their autophagy. J Cell Biol.

[50] Callegari S, Oeljeklaus S, Warscheid B, Dennerlein S, Thumm M, Rehling P et al. Phospho-ubiquitin-PARK2 complex as a marker for mitophagy defects. Autophagy 2017;13:1254852. 10.1080/15548627.2016.1254852 PMC5240832 27846363

[51] Lazarou M, Sliter DA, Kane LA, Sarraf SA, Wang C, Burman JL (2015). The ubiquitin kinase PINK1 recruits autophagy receptors to induce mitophagy. Nature.

[52] Pickrell AM, Huang CH, Kennedy SR, Ordureau A, Sideris DP, Hoekstra JG et al. Endogenous Parkin Preserves Dopaminergic Substantia Nigral Neurons following Mitochondrial DNA Mutagenic Stress. Neuron 2015;87:034. 10.1016/j.neuron.2015.06.034 PMC4803114 26182419

[53] Sung H, Tandarich LC, Nguyen K, Hollenbeck PJ. Compartmentalized regulation of Parkin-mediated mitochondrial quality control in the Drosophila nervous system in vivo. J Neurosci 2016;36:2016. 10.1523/JNEUROSCI.0633-16.2016 PMC4945662 27413149

[54] Peker N, Donipadi V, Sharma M, McFarlane C, Kambadur R. Loss of Parkin impairs mitochondrial function and leads to muscle atrophy. Am J Physiol - Cell Physiol 2018;315:2017. 10.1152/ajpcell.00064.2017 29561660

[55] Suen DF, Narendra DP, Tanaka A, Manfredi G, Youle RJ. Parkin overexpression selects against a deleterious mtDNA mutation in heteroplasmic cybrid cells. Proc Natl Acad Sci USA 2010;107:914569. 10.1073/pnas.0914569107 PMC2900690 20547844

[56] Narendra D, Walker JE, Youle R. Mitochondrial quality control mediated by PINK1 and Parkin: Links to parkinsonism. Cold Spring Harb. Perspect. Biol. 2012;4:a011338. 10.1101/cshperspect.a011338 PMC3536340 23125018

[57] Chen Z, Liu L, Cheng Q, Li Y, Wu H, Zhang W (2017). Mitochondrial E3 ligase MARCH 5 regulates FUNDC 1 to fine‐tune hypoxic mitophagy. EMBO Rep.

[58] Yun J, Puri R, Yang H, Lizzio MA, Wu C, Sheng Z-H et al. MUL1 acts in parallel to the PINK1/parkin pathway in regulating mitofusin and compensates for loss of PINK1/parkin. Elife 2014:e01958. 10.7554/eLife.01958 PMC4044952 24898855

[59] Strappazzon F, Nazio F, Corrado M, Cianfanelli V, Romagnoli A, Fimia GM et al. AMBRA1 is able to induce mitophagy via LC3 binding, regardless of PARKIN and p62/SQSTM1. Cell Death Differ. 2015;22:139. 10.1038/cdd.2014.190 PMC4326578 25661525

[60] Liu L, Feng D, Chen G, Chen M, Zheng Q, Song P (2012). Mitochondrial outer-membrane protein FUNDC1 mediates hypoxia-induced mitophagy in mammalian cells. Nat Cell Biol.

[61] Erbil-Bilir S, Kocaturk NM, Yayli M, Gozuacik D. Study of protein-protein interactions in autophagy research. J Vis Exp. 2017;2017:55881. 10.3791/55881 PMC5752206 28930972

[62] Demirbag-Sarikaya S, Akkoc Y, Turgut S, Erbil-Bilir S, Kocaturk NM, Dengjel J (2022). A novel ATG5 interaction with Ku70 potentiates DNA repair upon genotoxic stress. Sci Rep..

[63] Bayraktar O, Oral O, Kocaturk NM, Akkoc Y, Eberhart K, Kosar A et al. IBMPFD disease-causing mutant VCP/p97 proteins are targets of autophagic-lysosomal degradation. PLoS One. 2016; 11:164864. 10.1371/journal.pone.0164864 PMC5074563 27768726

[64] Yazgili AS, Meul T, Welk V, Semren N, Kammerl IE, Meiners S. In-gel proteasome assay to determine the activity, amount, and composition of proteasome complexes from mammalian cells or tissues. STAR Protoc. 2021;2:100526. 10.1016/j.xpro.2021.100526 PMC8121766 34027484

[65] Antonioli M, Ciccosanti F, Dengjel J, Fimia GM. Methods to Study the BECN1 Interactome in the Course of Autophagic Responses. *Methods Enzymol*. 2017:429–45. 10.1016/bs.mie.2016.09.069 28253970

[66] Krzywinski M, Altman N (2013). Points of significance: Power and sample size. Nat Methods.

