# Supplementary material for: Novel protein complexes containing autophagy and UPS components regulate proteasome-dependent PARK2 recruitment onto mitochondria and PARK2-PARK6 activity during mitophagy
Source: Cell Death Dis. 2022 Nov 10;13(11):947. doi: 10.1038/s41419-022-05339-x (PMC9649694; doi:10.1038/s41419-022-05339-x)
Supplement: Supplementary file 1 — Supplementary Figure Legends [file 41419_2022_5339_MOESM1_ESM.docx]

**Supplementary Figure Legends**

**Figure S1.** PSMA7 is a novel ATG5 interactor. *A,* Representative western blot image showing PSMA7 and ATG5 protein levels in total cell lysate (Input) and FLAG-immunoprecipitated lysate (FLAG-IP) of HEK293T cells transfected with FLAG-PSMA7 and/or non-tagged hATG5. Actin beta (ACTB) was used as loading control. Band intensity ratios were provided below the image. *B,* Representative western blot image showing ATG5 and PSMA7 protein levels in total (Input) and FLAG-immunoprecipitated (FLAG-IP) lysate of HEK293T cells transfected with FLAG-PSMA7 and non-tagged hATG5 constructs, and treated with CCCP (10 μM) or staurosporine (STAURO, 1 μM) for 12 h. ACTB was used as loading control. Band intensity ratios were indicated below. *C,* Representative western blot images of ATG5-12 and PSMB5 proteins in total (Input) and FLAG-immunoprecipitated (FLAG-IP) cell lysate isolated from HEK293T cells transfected with FLAG-ATG5 and treated with either DMSO or CCCP (10 μM) for 12 h. ACTB was used as loading control. Band intensity ratios were indicated below the image. *D*, Representative western blot images of endogenous PARK2 and ATG5-12 proteins in total (Input) and ATG5-immunoprecipitated (ATG5-IP) cell lysate isolated from HT-22 cells treated with either DMSO or CCCP (10 μM) for 12 h. ACTB was used as loading control. *E*, Representative western blot images of endogenous PARK2 and ATG5-12 proteins in total (Input) and ATG5-immunoprecipitated (ATG5-IP) cell lysate isolated from SH-SY5Y cells treated with either DMSO or CCCP (10 μM) for 12 h. ACTB was used as loading control. *F* and *H*, Representative confocal images of HeLa cells co-transfected with GFP-PSMA7 along with pmCherry-ATG5 (*G*) or GFP-PSMA7 and cherry-PARK2 (*I*) and treated with DMSO, CCCP (20 μM, 2 h) or O/A (10 μM, 2 h). PSMA7 cellular distribution analysed under a confocal microscope. *G* and *I*, Quantification graphs showing the cellular pattern of GFP-PSMA7 (Mean ± S.D. of three independent experiments, n=60, ordinary one-way ANOVA)

**Figure S2.** Autophagy and UPS components, including cleaved/processed PARK6, are found in total cell lysates and in the cytosolic fractions of cells. *A*, *C*, *E* and *G*, Western blot images representing the levels of PARK2, PARK6, PSMA7 and ATG5-12 following gel filtration chromatography performed using total (*A* and *C*) and cytosolic (*E* and *G*) fraction isolated from YFP-PARK2 expressing HEK293T cells treated with either DMSO (*A* and *E)*, or CCCP (*C* and *G)* for 12 h. L, Lysate. L-PARK6, uncleaved/unprocessed form of PARK6 (63 kDa). S-PARK6, cleaved/processed form of PARK6 (55 kDa). *B*, *D*, *F* and *H*, Graphs representing PARK2 protein levels quantified from *A*, *C*, *E* and *G* respectively (mean±S.D., n=3).

**Figure S3.** Inhibition of proteasome activity by drugs leads to attenuation of PARK2 translocation onto mitochondria. *A*, Representative western blot images of P27 and CCND1 in HEK 293T cells treated with DMSO or MG132 (30 µM, 2.5 or 12.5 h). Actin beta (ACTB) was used as loading control. *B,* Representative western blot images showing PARK2, ATG5-12, VDAC1 and ACTB protein levels in cytosolic (Cytosol) and mitochondrial (Mito) fractions of HA-PARK2 expressing HEK293T cells treated with DMSO or CCCP (20 μM) for 2 h in the presence or absence of MG132 (30 μM). *C*, Graph representing the quantification graph representing mitochondrial PARK2 level as normalized to mitochondrial loading control VDAC1 (mean±S.D., n=3). *D,* Representative western blot images showing PARK2, ATG5-12, VDAC1 and ACTB protein levels in cytosolic (Cytosol) and mitochondrial (Mito) fractions of HA-PARK2 expressing HEK293T cells treated with DMSO or CCCP (20 μM) for 2 h in the presence or absence of Bortezomib (30 μM). Band intensity ratios were provided below the image.

**Figure S4.** Inhibition of proteasome function does not alter turnover of PARK2 and ATG5-12. *A,* Representative western blot images of PARK6, PARK2 and ATG5-12 proteins in total cell lysate isolated from HEK293T cells transfected with MYC-PARK2 and GFP-PARK6 treated with CCCP (20 µM, 2 or 12 h) in the presence of absence of MG132 (30 µM, 2.5 or 12.5 h). Actin beta (ACTB) was used as loading control. *B*, *C* and *D*, Graphs representing quantifications of PARK6 (*B*), PARK2 (*C*) and ATG5-12 (*D*) protein levels from *A* (mean±SEM, n=3).

**Figure S5.** CALR and TIM23 protein levels in mitochondrial fractions. *A*, *B*, *C* and *D*, Representative western blot images of ER protein CALR and mitochondrial protein TIM23 in subcellular fractions isolated in Fig. 2, C (*A*), Fig. 4, A (*B*), Fig. 5, A (*C*) and Fig. 7, A (*D*). Total indicates total cell lysate.

**Figure S6.** Knockdown of PSMA7 impairs mitophagy. *A,* Representative confocal images of MYC- PARK2, GFP- optineurin (OPTN, green) and mito-dsRed (red) expressing HeLa cells transfected with non-targeting control siRNA (siCNT) or PSMA7-targeting siRNA (siP7), and treated with DMSO or CCCP (10 μM) for 12 h. MERGE, overlay of green and red signals. *B,* Graphs showing percentage of cells detected with mitochondria associated OPTN dots (mean±S.D., n=3). *C,* Confocal images representing mitochondrial DNA (mtDNA, green) and nuclear DNA (nDNA, blue) in MYC-PARK2 expressing HeLa cells transfected with either siCNT or siP7, and treated with DMSO or CCCP (10 μM) for 12 h. *D,* Graph showing the ratio of mtDNA/nDNA from *C* (mean±S.D., n=3).

**Figure S7.** Uncropped western blot images of Fig. 1A, 1C, 1E, 1G and 1I

**Figure S8.** Uncropped western blot images of Fig. 2A, 2C, 3A and 3C

**Figure S9.** Uncropped western blot images of Fig. 4A, 5A and 6A

**Figure S10.** Uncropped western blot images of Fig. 7A, 7E, 7F, 7G and 7H

**Figure S11.** Uncropped western blot images of Fig. 8A, 8E and 8G

**Figure S12.** Uncropped western blot images of Fig. S1A, S1B, S1C, S1D and S1E

**Figure S13.** Uncropped western blot images of Fig. S2A, S2C, S2E and S2G

**Figure S14.** Uncropped western blot images of Fig. S3A, S3B, S3D and S4A

**Figure S15.** Uncropped western blot images of Fig. S5A, S5B, S5C and S5D
