# Supplementary figures and images for: Novel protein complexes containing autophagy and UPS components regulate proteasome-dependent PARK2 recruitment onto mitochondria and PARK2-PARK6 activity during mitophagy
Source: Cell Death Dis. 2022 Nov 10;13(11):947. doi: 10.1038/s41419-022-05339-x (PMC9649694; doi:10.1038/s41419-022-05339-x)

Fig. S1

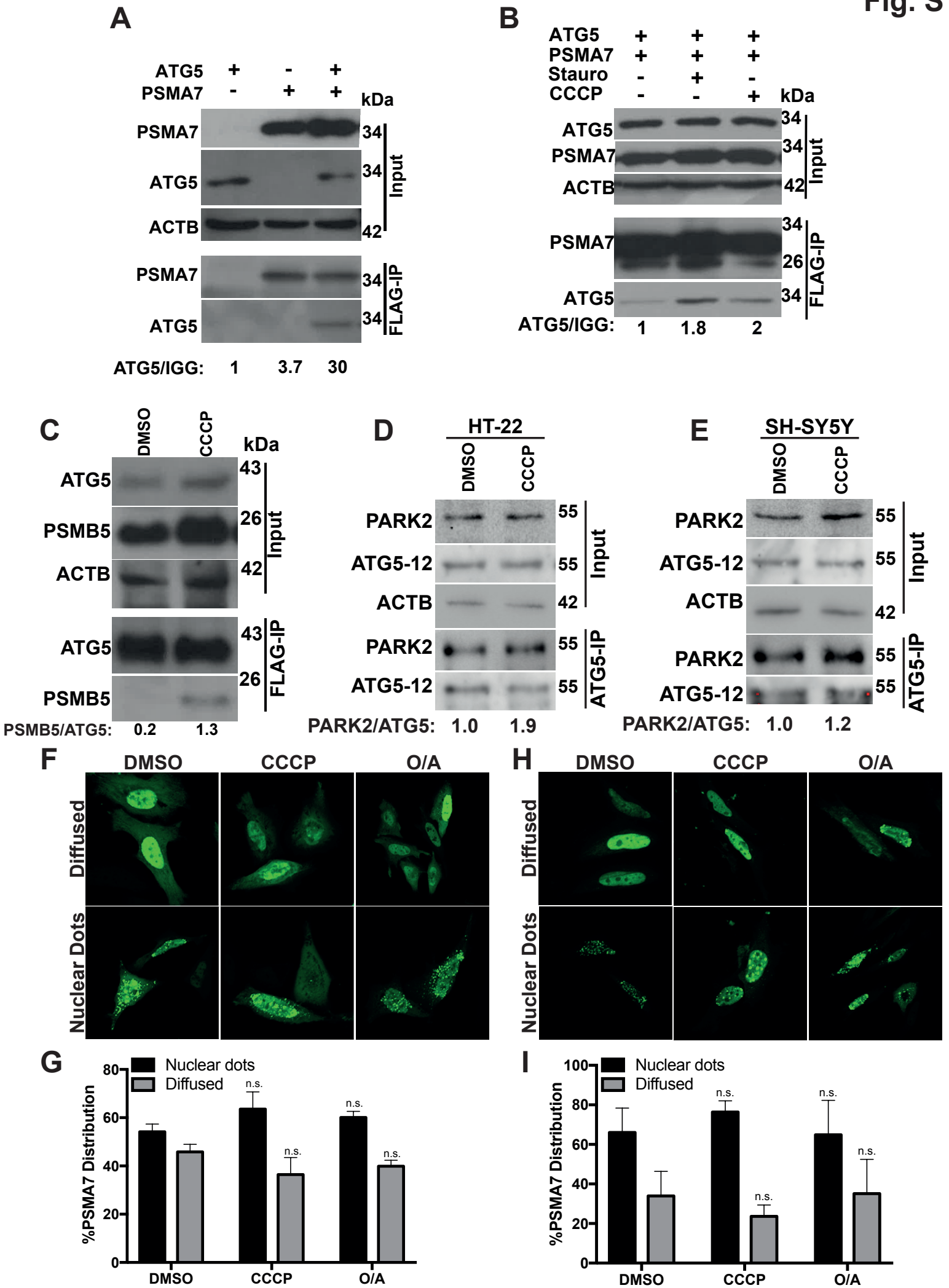

Supplement: Supplementary file 2 — Figure S1 [file 41419_2022_5339_MOESM2_ESM.pdf]

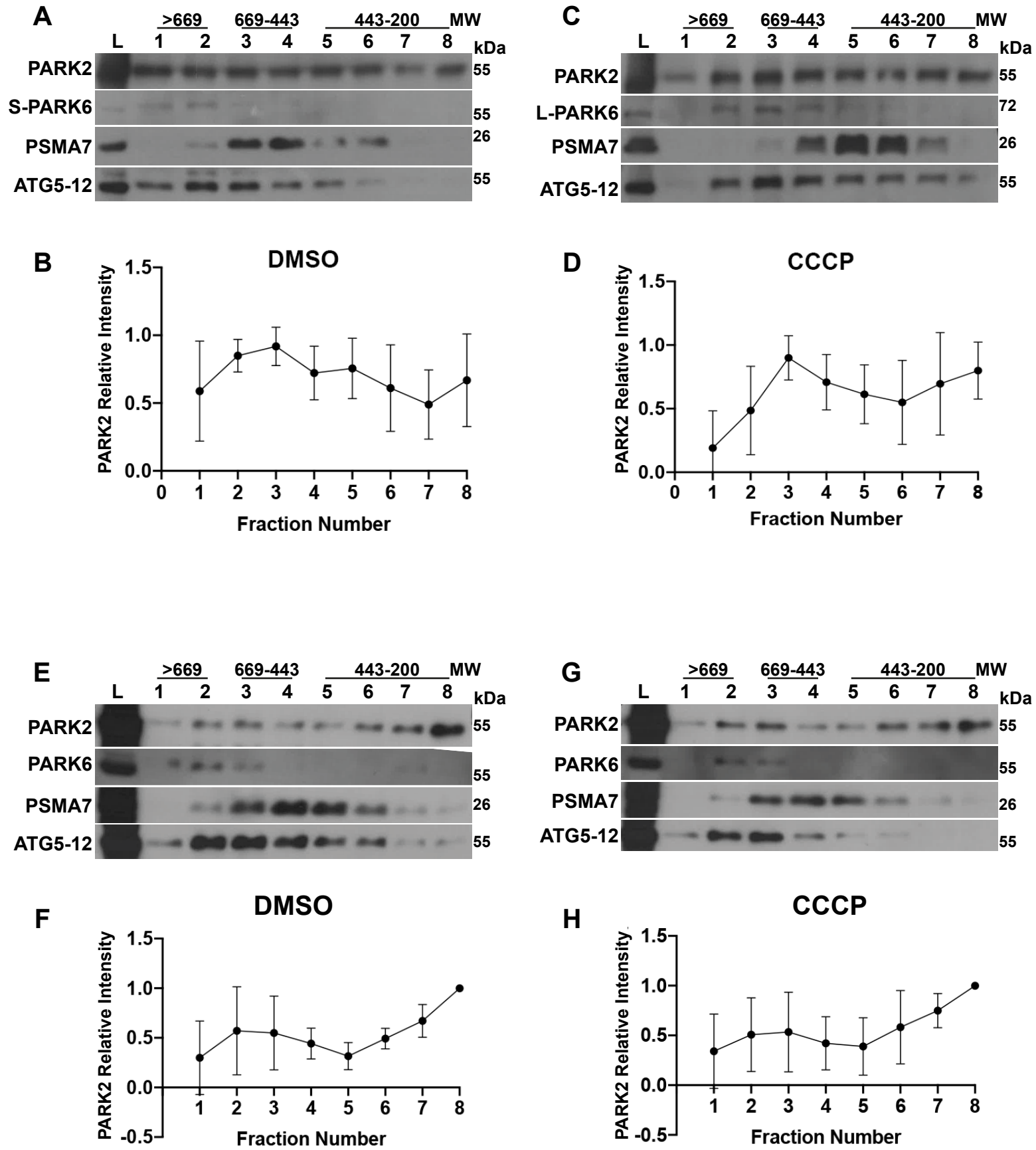

Supplement: Supplementary file 3 — Figure S2 [file 41419_2022_5339_MOESM3_ESM.pdf]

**A**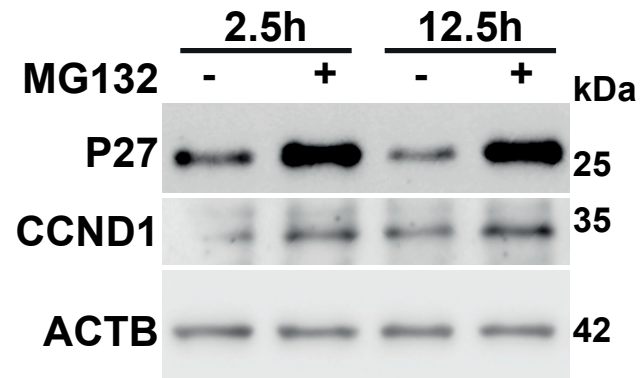**B**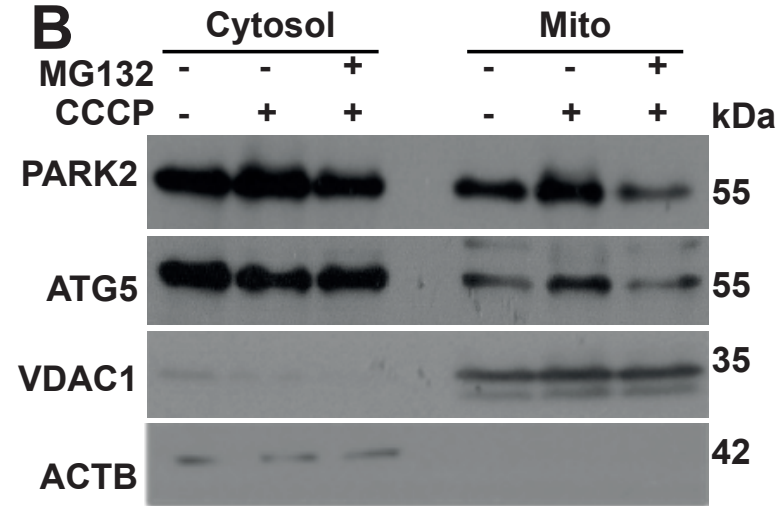**C**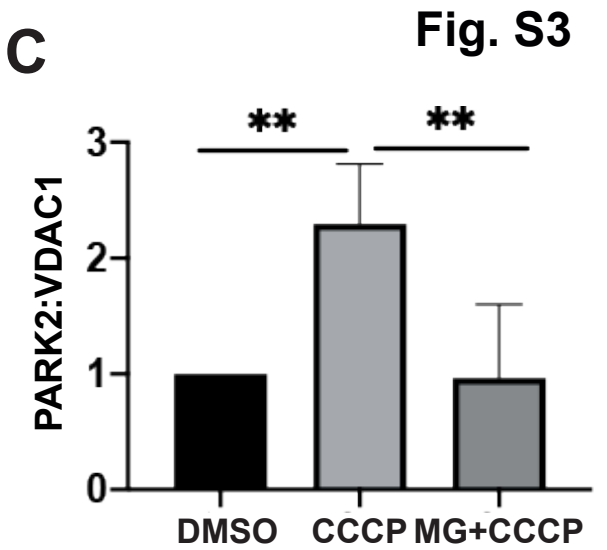**D**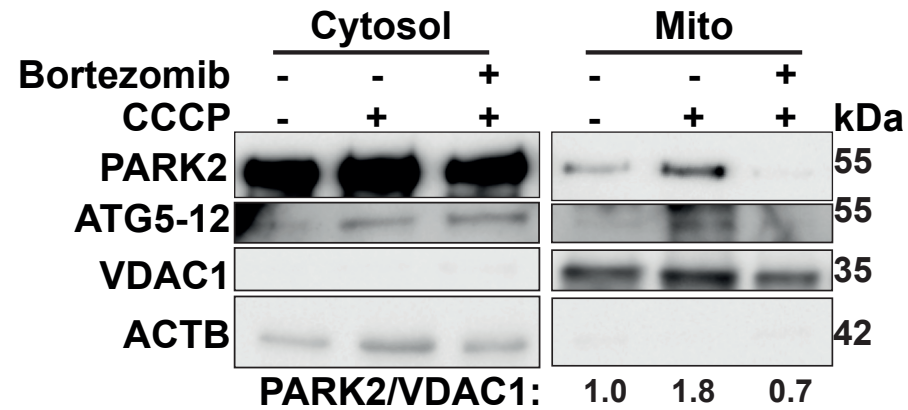

Supplement: Supplementary file 4 — Figure S3 [file 41419_2022_5339_MOESM4_ESM.pdf]

Fig. S4

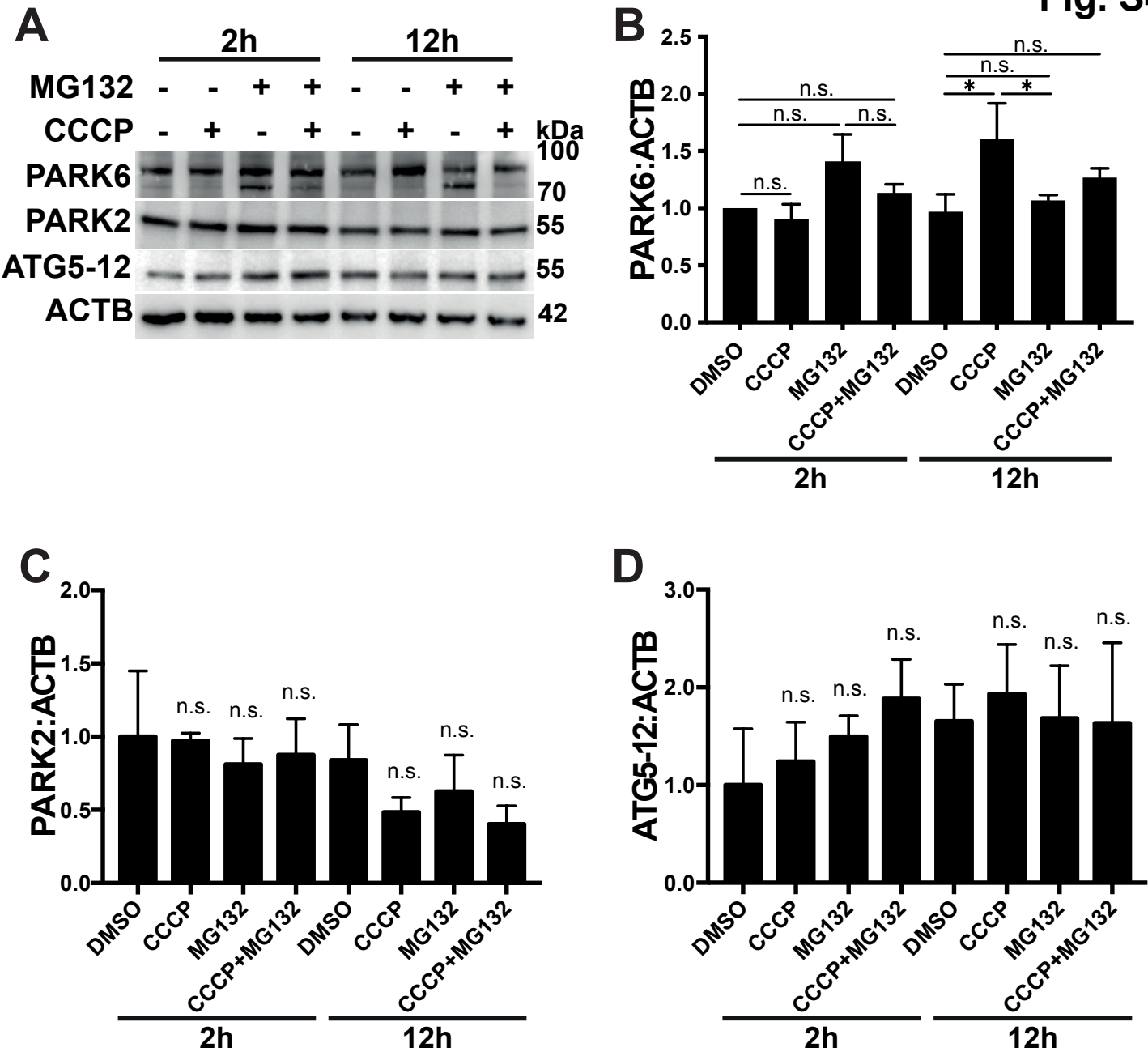

Supplement: Supplementary file 5 — Figure S4 [file 41419_2022_5339_MOESM5_ESM.pdf]

**Fig. S5**

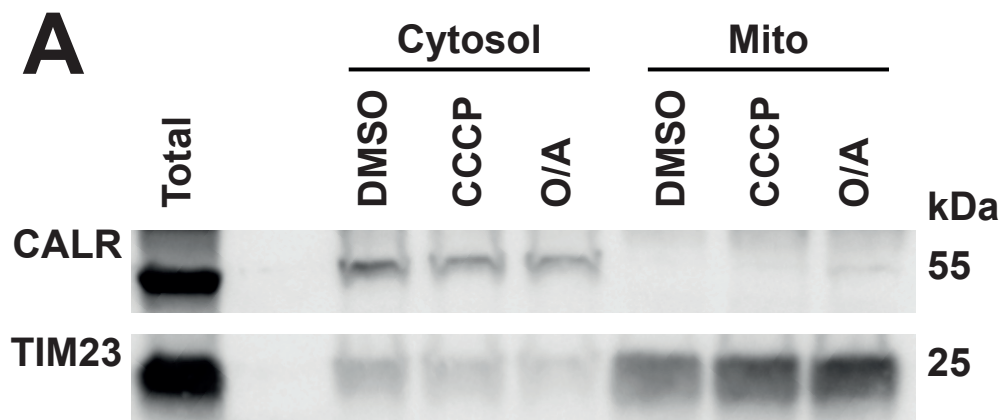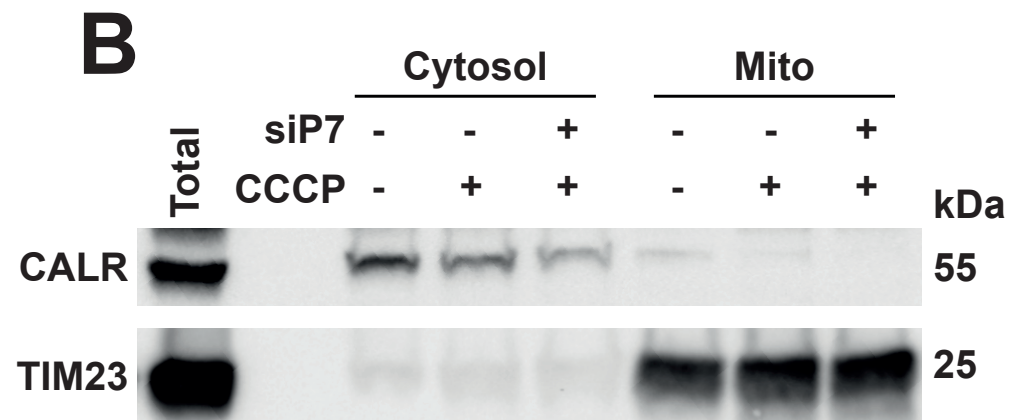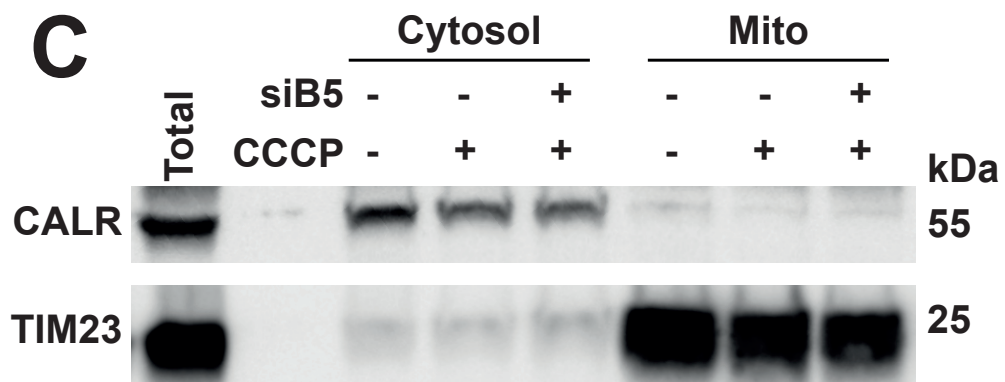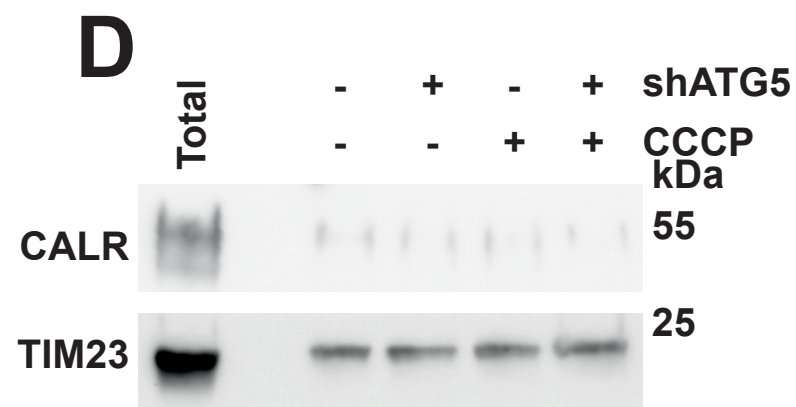

Supplement: Supplementary file 6 — Figure S5 [file 41419_2022_5339_MOESM6_ESM.pdf]

Fig. S6

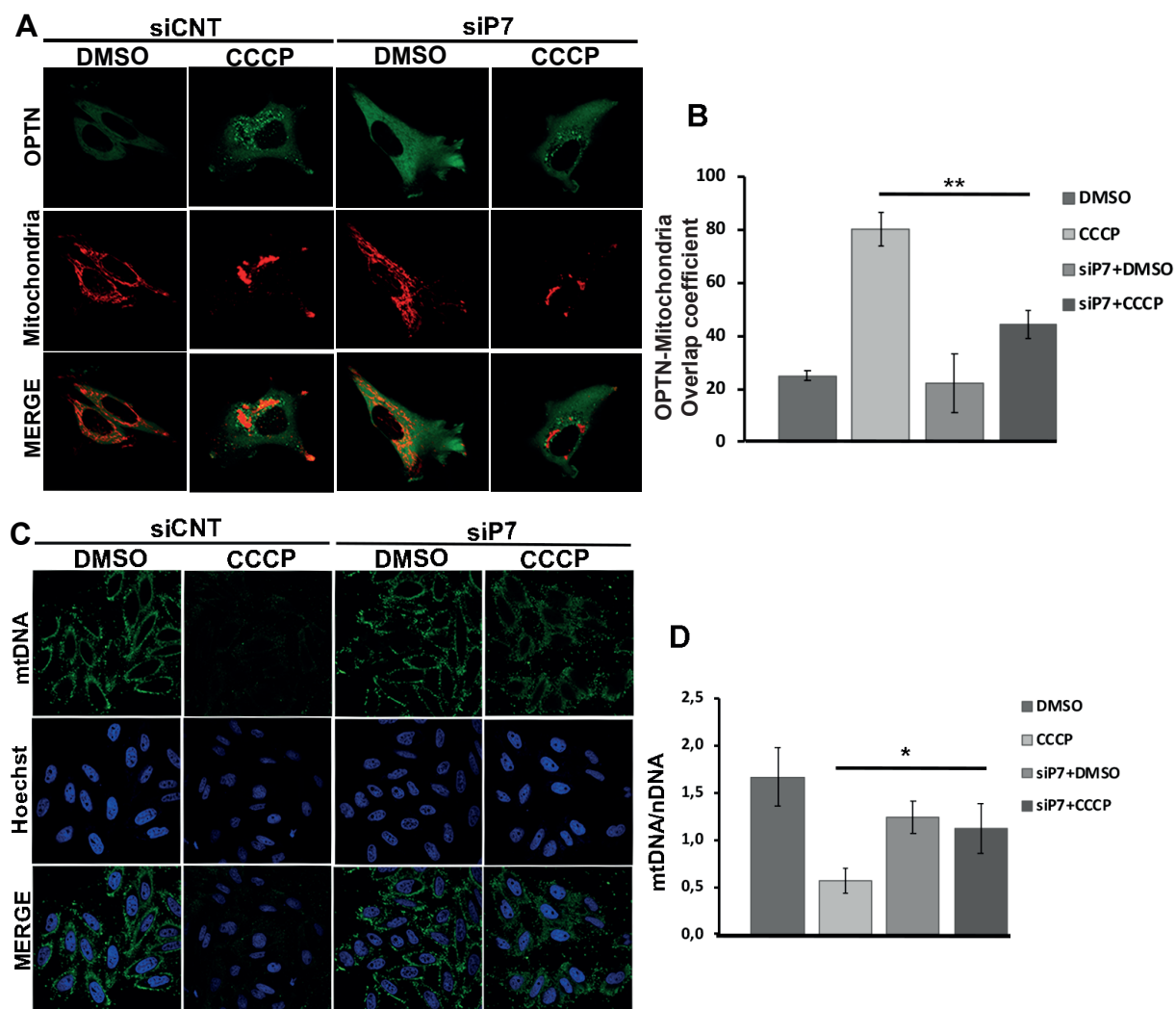

Supplement: Supplementary file 7 — Figure S6 [file 41419_2022_5339_MOESM7_ESM.pdf]

**Fig. S7**

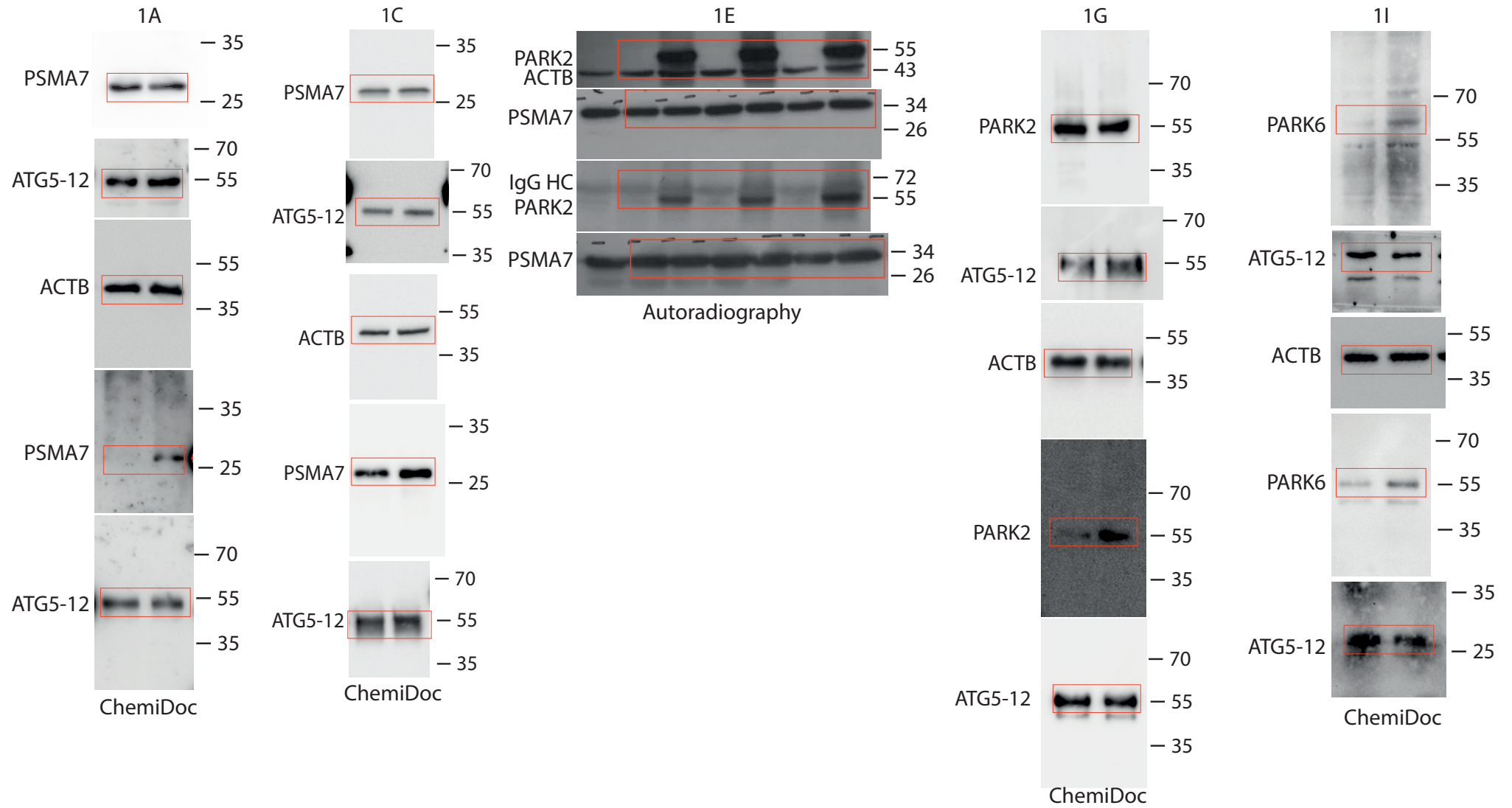

Supplement: Supplementary file 8 — Figure S7 [file 41419_2022_5339_MOESM8_ESM.pdf]

**Fig. S8**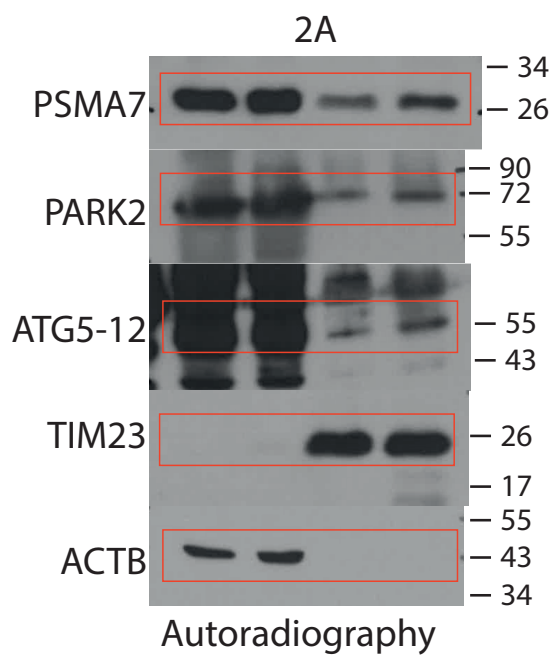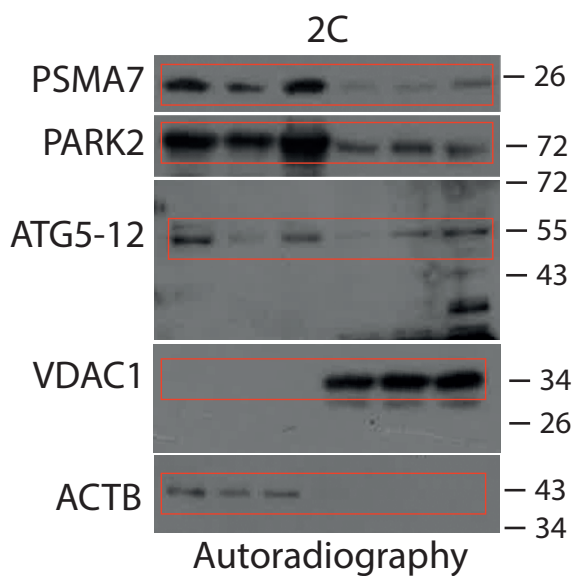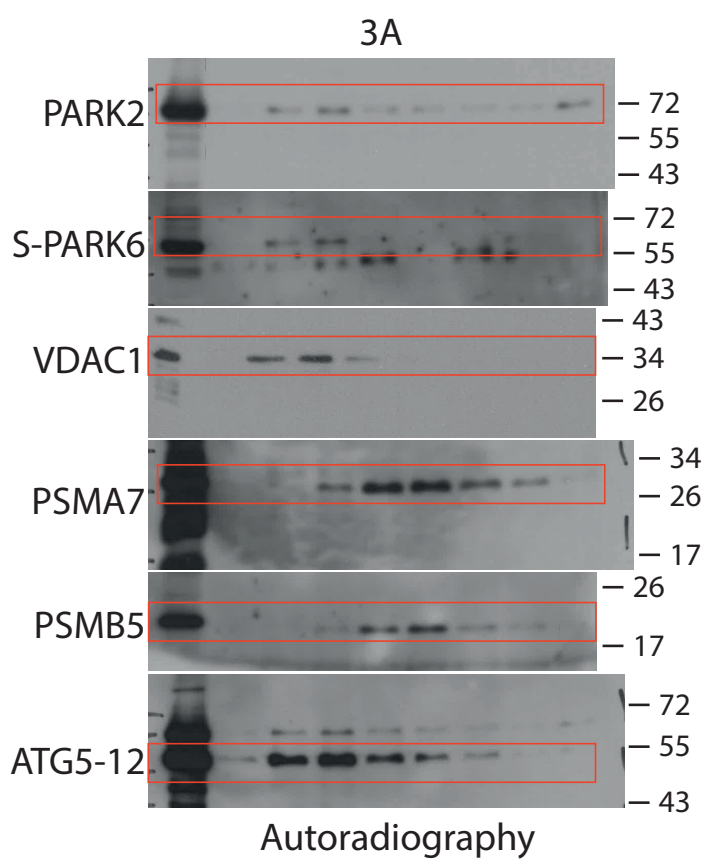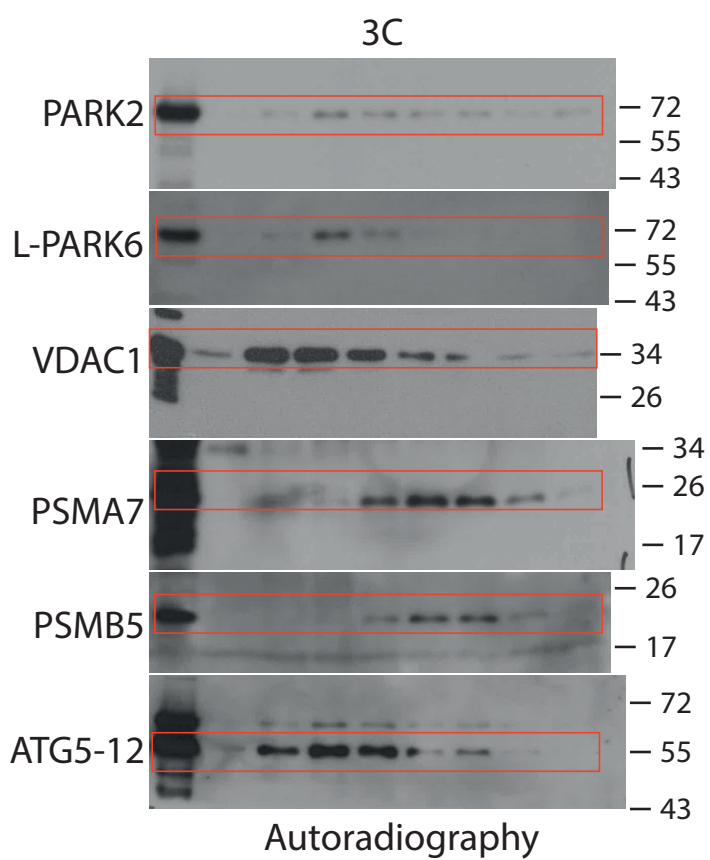

Supplement: Supplementary file 9 — Figure S8 [file 41419_2022_5339_MOESM9_ESM.pdf]

**Fig. S9**

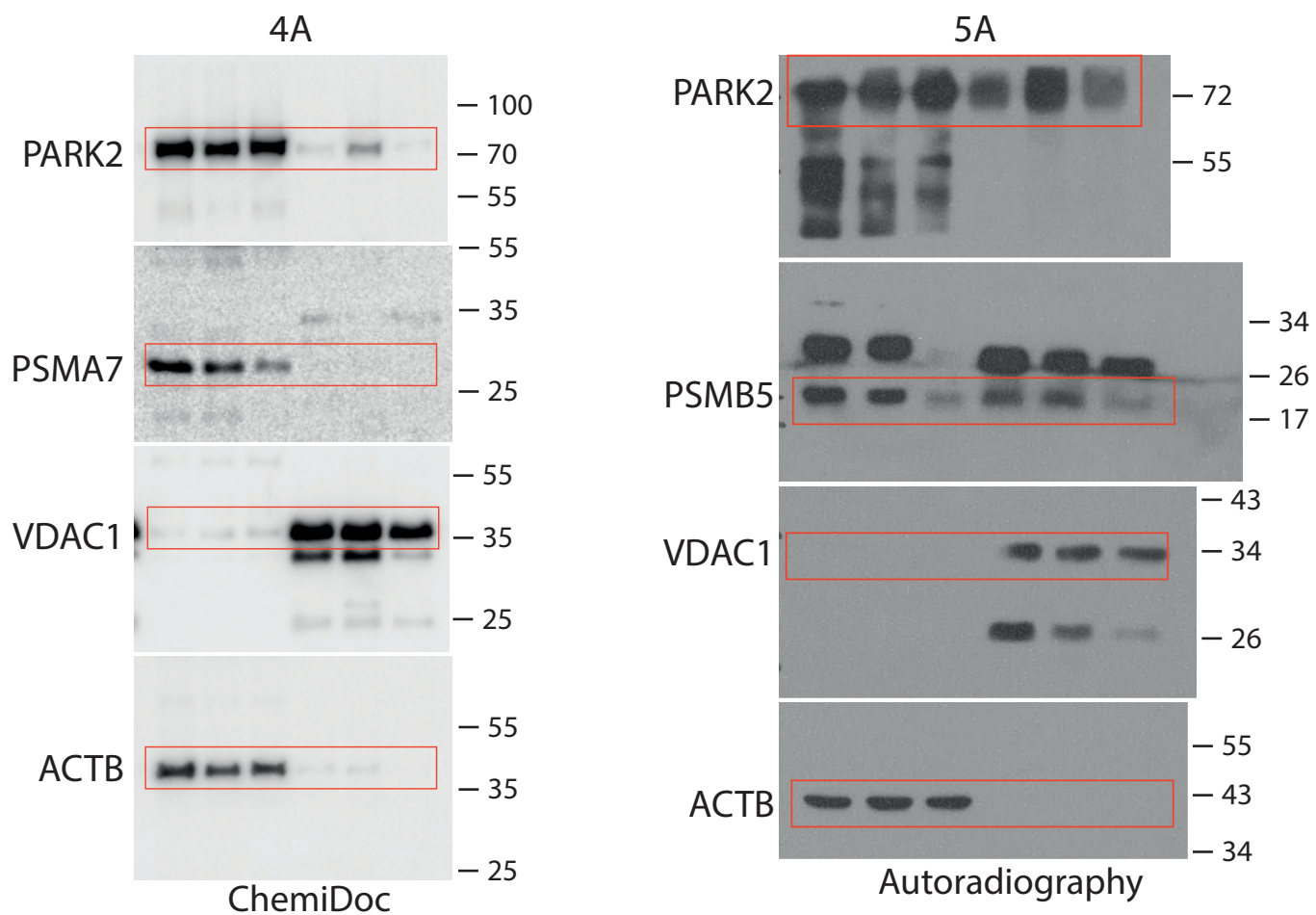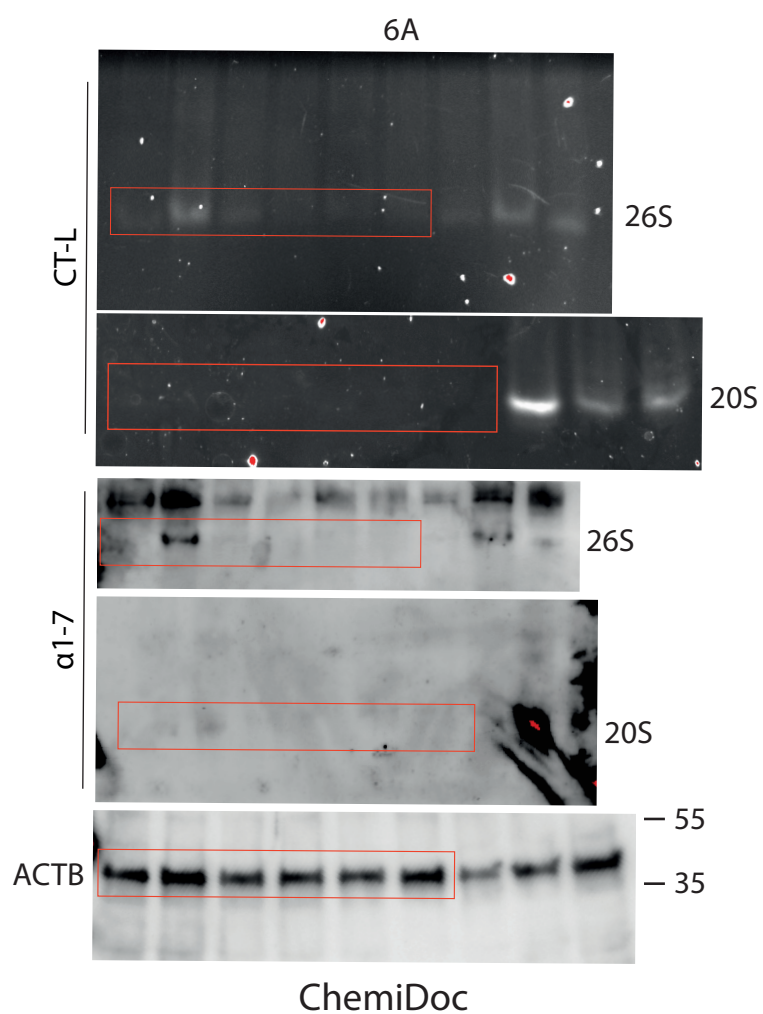

Supplement: Supplementary file 10 — Figure S9 [file 41419_2022_5339_MOESM10_ESM.pdf]

**Fig. S10**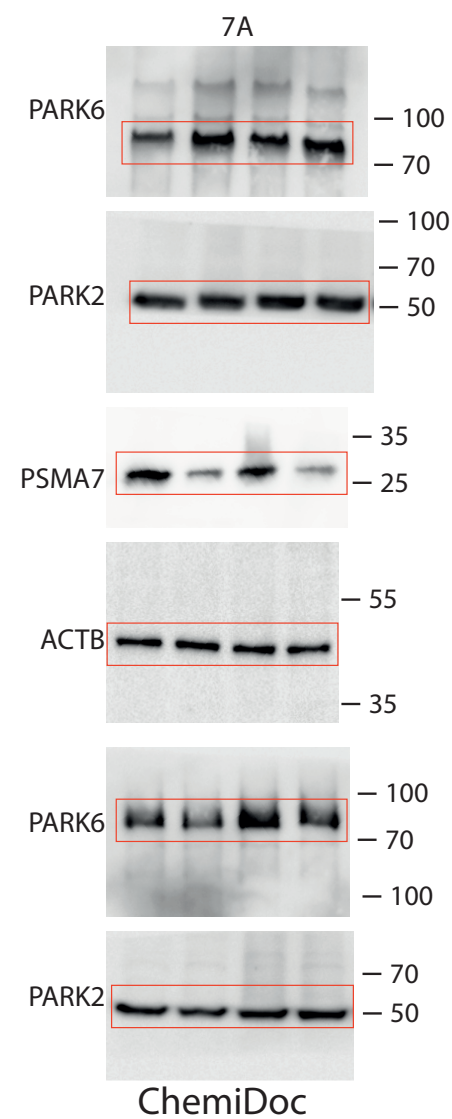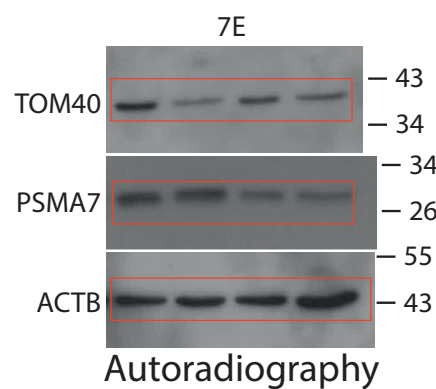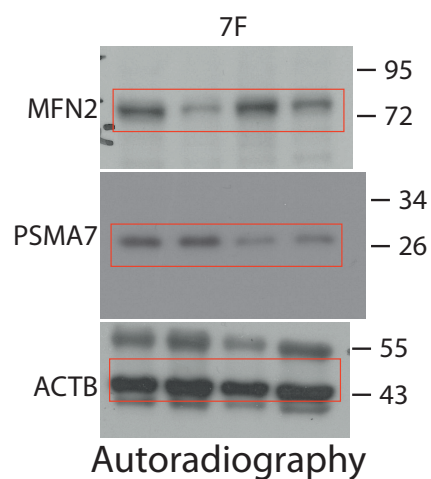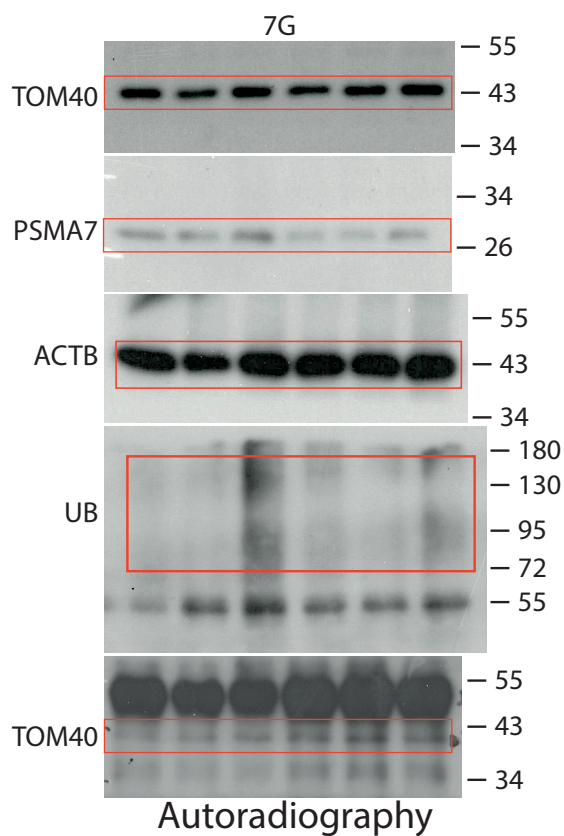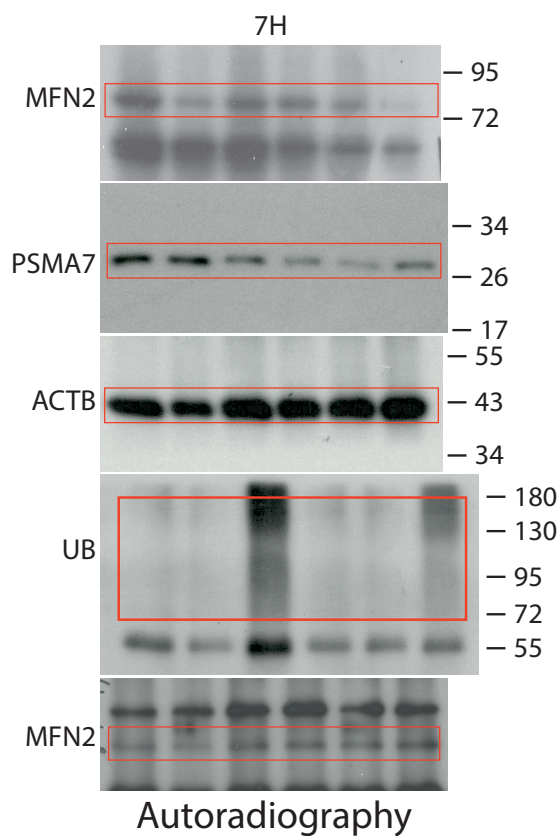

Supplement: Supplementary file 11 — Figure S10 [file 41419_2022_5339_MOESM11_ESM.pdf]

**Fig. S11**

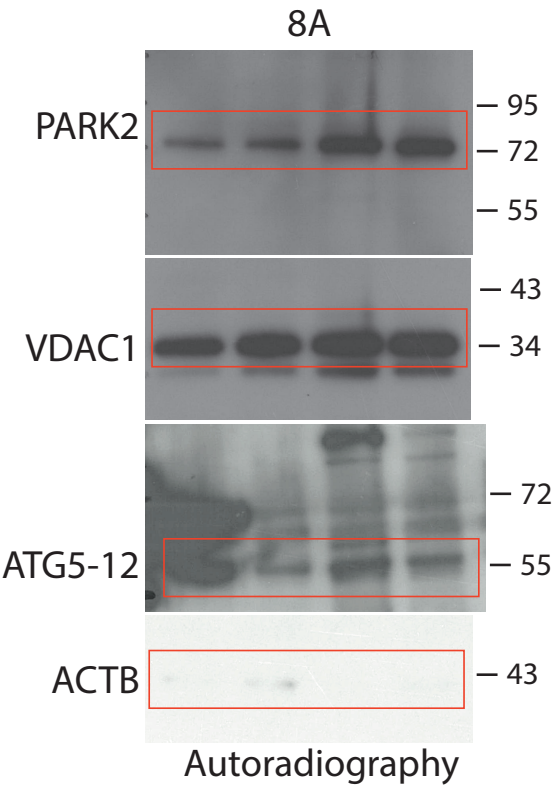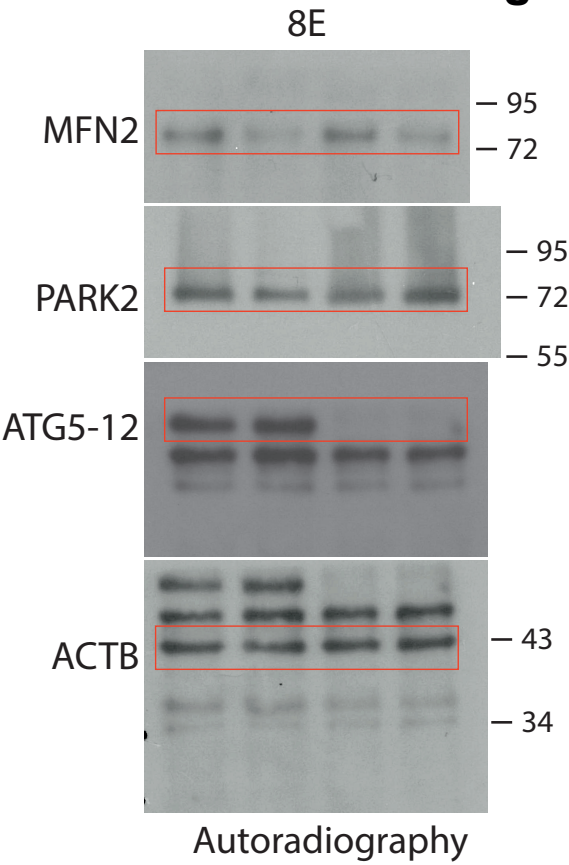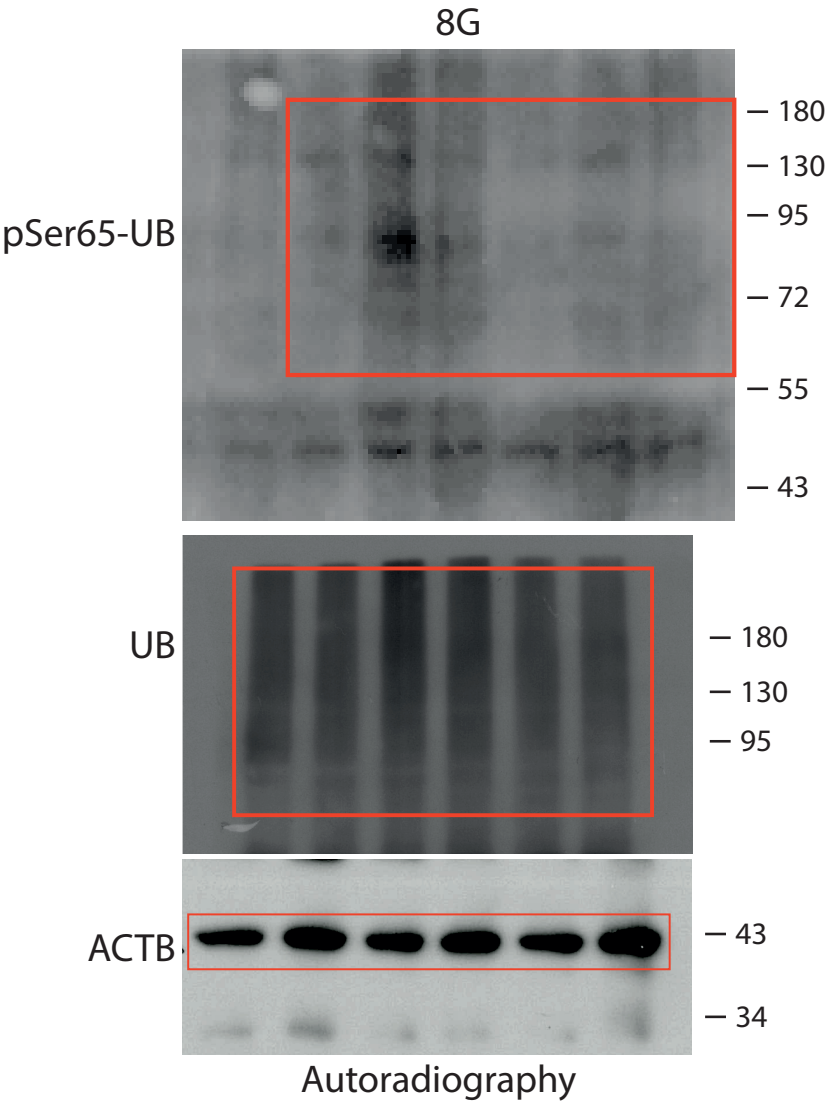

Supplement: Supplementary file 12 — Figure S11 [file 41419_2022_5339_MOESM12_ESM.pdf]

**Fig. S12**

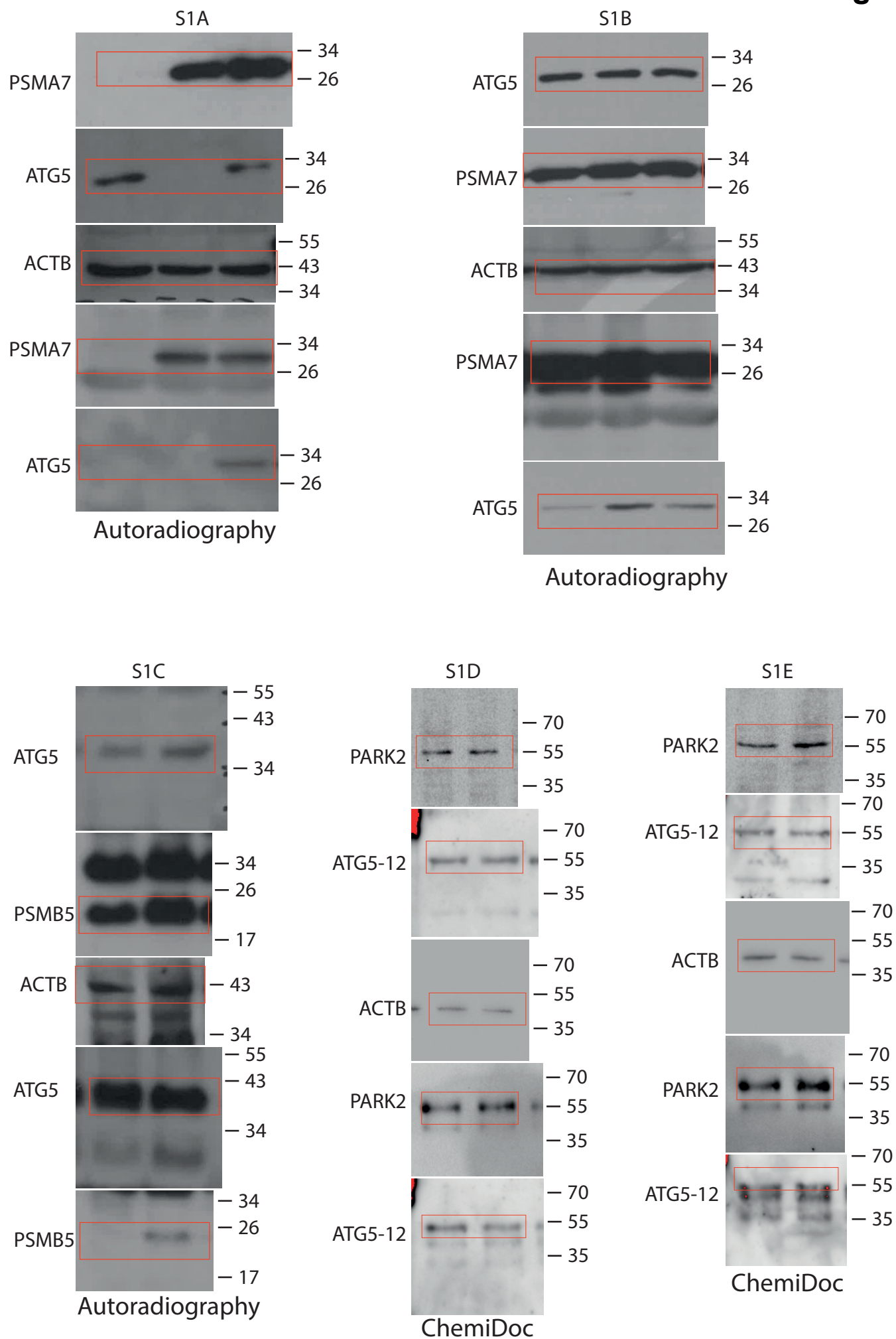

Supplement: Supplementary file 13 — Figure S12 [file 41419_2022_5339_MOESM13_ESM.pdf]

**Fig. S13**

S2A

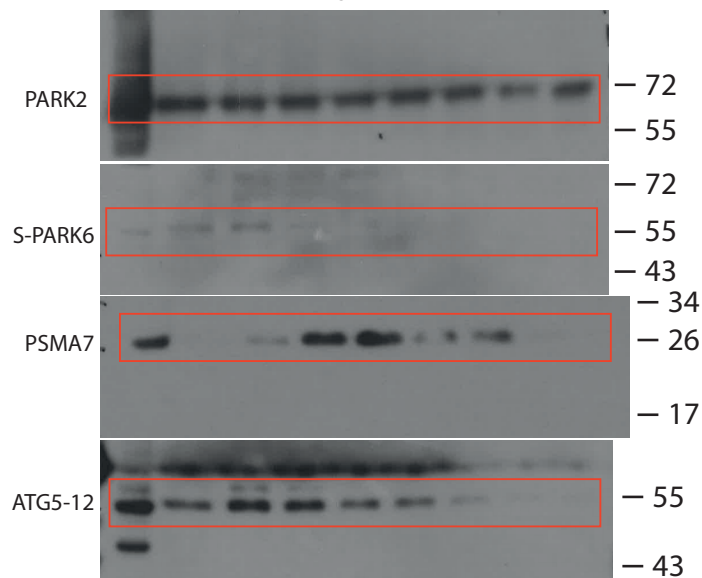

Autoradiography

S2C

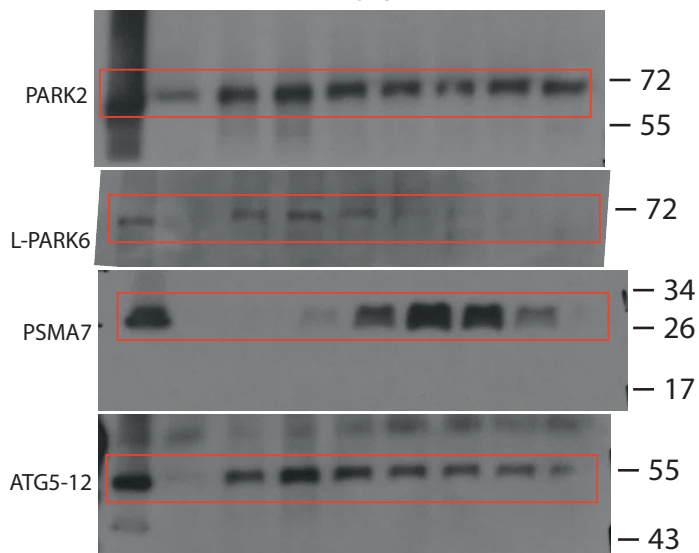

Autoradiography

S2E

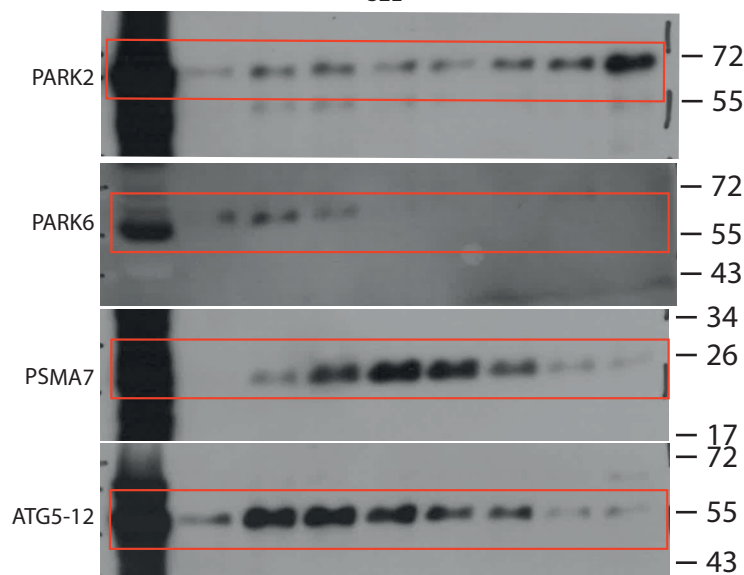

Autoradiography

S2G

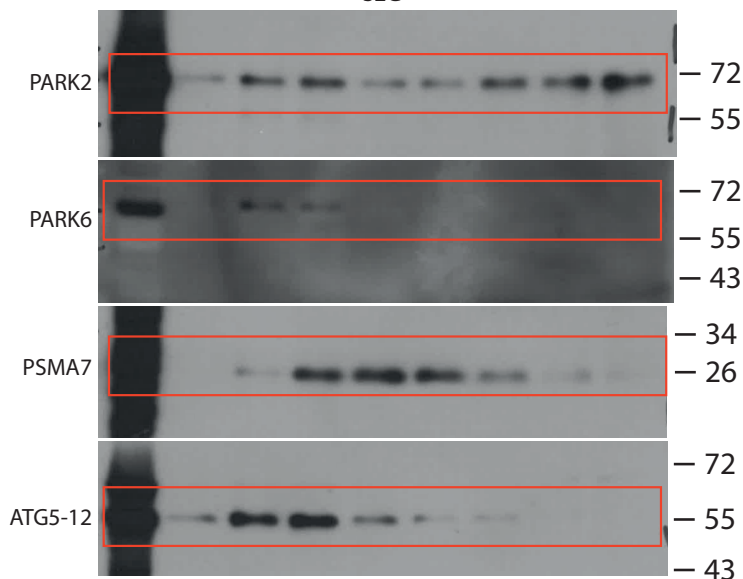

Autoradiography

Supplement: Supplementary file 14 — Figure S13 [file 41419_2022_5339_MOESM14_ESM.pdf]

**Fig. S14**

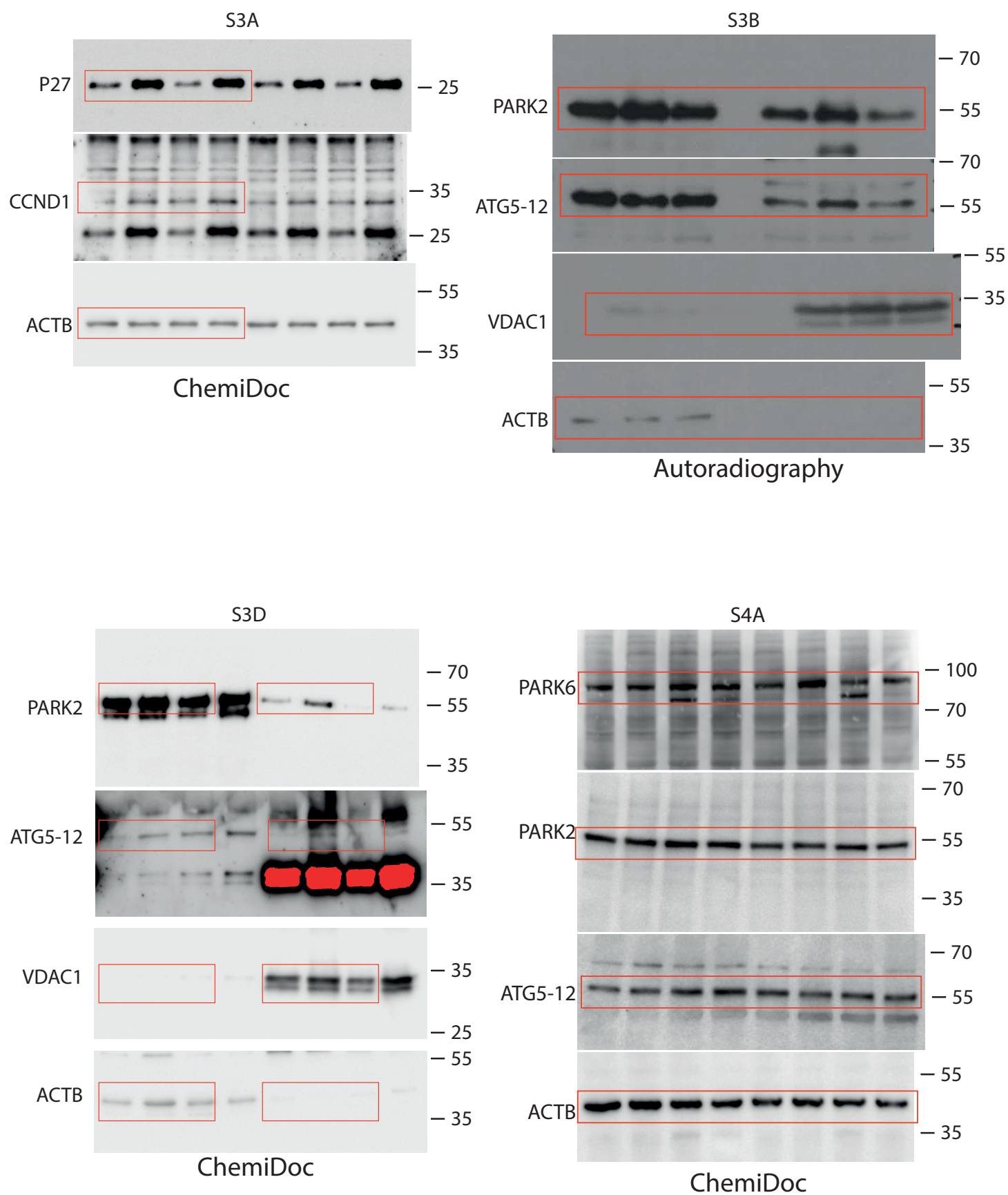

Supplement: Supplementary file 15 — Figure S14 [file 41419_2022_5339_MOESM15_ESM.pdf]

**Fig. S15**

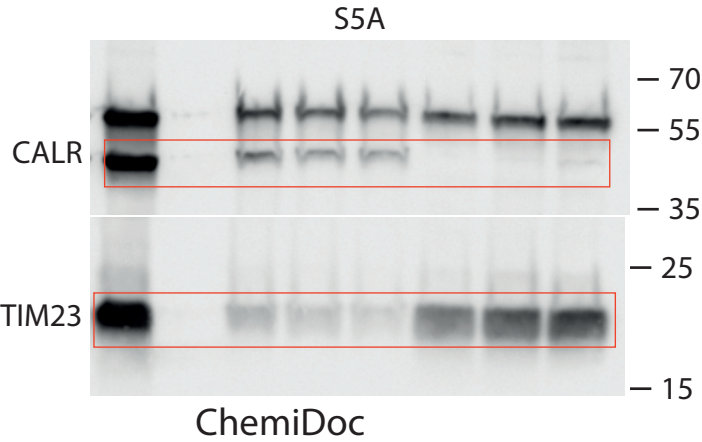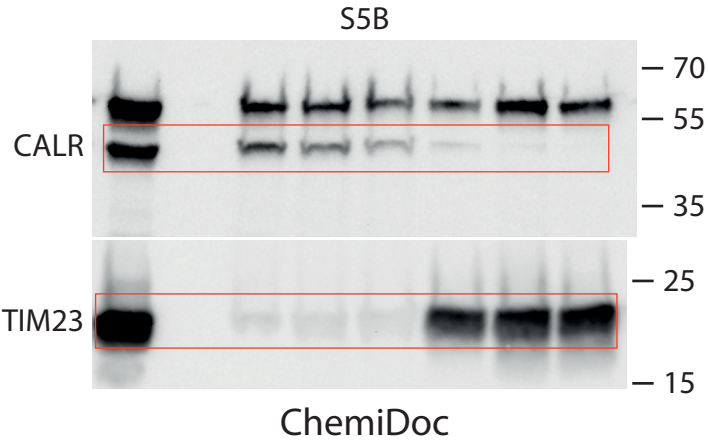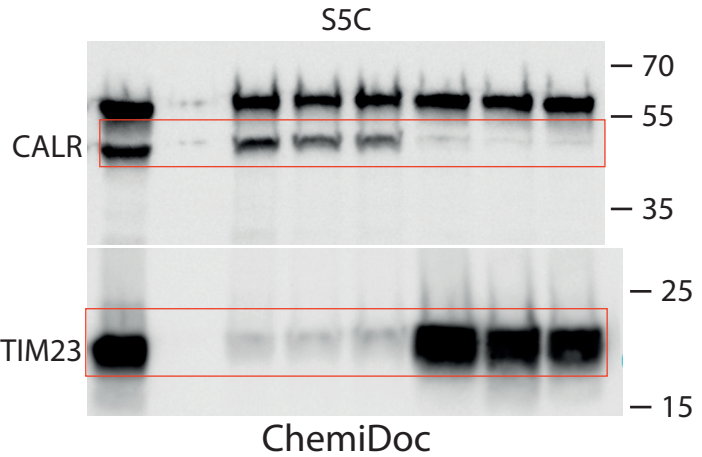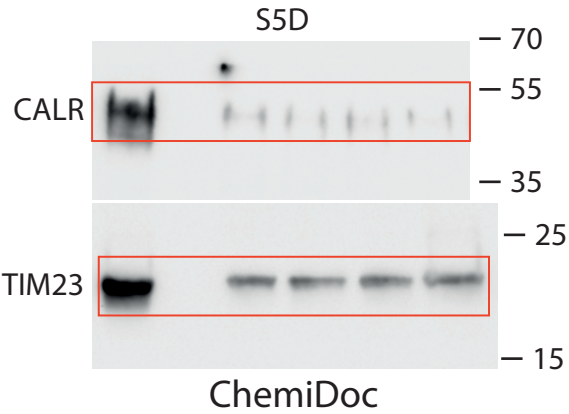

Supplement: Supplementary file 16 — Figure S15 [file 41419_2022_5339_MOESM16_ESM.pdf]
